# Biomimetic all-metal Pd_11_ helicene

**DOI:** 10.1126/sciadv.aef7488

**Published:** 2026-07-15

**Authors:** Yan Sun, Shuguang Wang, Jiachun Li, Baoliang Han, Zibao Gan, Xiuwen Zheng, Di Sun

**Affiliations:** ^1^ Key Laboratory of Advanced Biomaterials and Nanomedicine in Universities of Shandong, Linyi University, Linyi 276000, China.; ^2^ School of Chemistry and Chemical Engineering, Qilu Normal University, Jinan 250013, China.; ^3^ School of Chemistry and Chemical Engineering, Shandong University, Jinan 250100, China.

## Abstract

Since their discovery in the early 20th century, helicenes have emerged as a distinctive class of polycyclic aromatic compounds with rigid, screw-shaped chiral frameworks and substantial photoelectrical properties. Over the past century, this family has expanded to include diverse heterohelicenes and functional derivatives. In sharp contrast, the rational construction of all-metal helicenes, in which the helical backbone is entirely composed of metal elements, remains a formidable synthetic challenge because of the difficulty of simultaneously controlling metal coordination geometry, helical propagation, and configurational stability. Herein, we report a stibine/thiolate–protected metallic helicene, Pd_11_(PhSb)_2_(AdmS)_10_, denoted as an antimony (Sb)–centered [3]Pd-helicene cluster. The Pd_11_ framework features a Pd_3_ triangle ortho-fused with two μ_6_-Sb–centered Pd_6_ rings, giving rise to an unprecedented slightly open-mouthed, scallop-like all-metal helicene architecture. Two 7-center-2-electron (7c-2e) σ bonds are localized on the Sb-centered Pd_6_ rings, stabilizing the helical all-metal skeleton. This metallic helicene can undergo structural “unfolding” through complete opening of the fused Pd_6_ rings, affording a chiral homolog, Pd_11_SCl(PhSb)_2_(AdmS)_11_. Notably, the Sb-centered [3]Pd-helicene cluster exhibits an exceptional electrocatalytic activity for two-electron oxygen reduction reaction, the reason for which was disclosed by operando infrared spectroscopy analysis and density functional theory calculations. This work represents an important advance in pursuit of all-metal helicene and opens avenues for their rational synthesis and functional exploitation.

## Teaser

An all-metal Pd helicene enables structural unfolding and efficient two-electron oxygen reduction performance.

## INTRODUCTION

Helicenes are a distinctive class of nonplanar polycyclic aromatic hydrocarbons that exhibit helical chirality arising from the ortho-fusion of arene or heteroarene rings (*1*–*6*). Their nomenclature uses either a bracketed number [*n*] or a Greek prefix to denote the number of fused aromatic units in the helical backbone; for example, pentahelicene is also denoted as [5]helicene (*7*). Owing to steric congestion between the terminal rings, an [*n*]helicene typically exists as a pair of stable enantiomers, designated *P* (right-handed) and *M* (left-handed) (*8*, *9*). The intrinsically chiral topology combined with extended π-conjugation endows helicenes with exceptional potential in asymmetric catalysis (*10*–*12*), biological sensing (*13*–*15*), organic optoelectronics (*16*–*20*), and molecular machines (*21*, *22*). Since the discovery of the first helicene in the early 20th century (*23*), this family has expanded to encompass carbohelicenes (*24*, *25*), heterohelicenes (*26*–*29*), multiple helicenes (*30*–*32*), organometallic helicenes (*33*, *34*), and a wide range of helicene derivatives (*35*, *36*). These advances have stimulated growing interest in the development of inorganic helicenes, helically chiral architectures in which the backbone is constructed from main-group or transition-metal elements. However, such species remain at a nascent stage, largely due to the intrinsic conflict between the nondirectional nature of metallic bonding and the stringent geometric requirements for enforcing a persistent helical topology.

The discovery of spherical carbon allotropes has long inspired chemists to pursue inorganic analogs on the basis of noncarbon elements. In recent decades, a variety of fullerene-like inorganic architectures have been realized, which can be broadly classified into two categories according to whether the cage framework is stabilized by external ligands or is composed solely of inorganic elements. One is the Zintl clusters without an external ligand, including the notable [Z@In_10_@Na_39_@In_74_] (Z = Ni, Pd, or Pt) (*37*), [As@Ni_12_@As_20_]^3−^ (*38*), [Sn@Cu_12_@Sn_20_]^12−^ (*39*), [Sb@Pd_12_@Sb_20_]^*n*−^ (*40*), [K@Au_12_Sb_20_]^5−^ (*41*), and [K_2_(Bi@Pd_12_@Bi_20_)]^4−^ (*42*). The other is the ligand-protected fullerene-like clusters, such as the “superfullerene” [Mo_132_O_372_(CH_3_CO_2_)_30_(H_2_O)_72_]^42−^ (*43*), spherical [{Cp*Fe(η^5^:η^1^:η^1^:η^1^:η^1^:η^1^-P_5_)}_12_{CuCl}_10_{Cu_2_Cl_3_}_5_{Cu(CH_3_CN)_2_}_5_] (*44*), golden fullerene Au_32_ (*45*), and Ag_135_Cu_60_ (*46*) cluster with an Ag_135_ fullerene-like topology. Collectively, these advances in inorganic fullerene chemistry provide substantial insights for the rational design of other inorganic analogs. Recently, You *et al.* (*47*) reported a mixed thiolate/phosphine–protected Pd_8_ cluster composed of two pseudocoplanar five-membered palladium (Pd) rings, reminiscent of a pentalene-like architecture. This result suggests that the ortho-fusion of multimembered Pd rings, stabilized by cooperative ligands, could represent a viable strategy to address the long-standing challenge of constructing metallic inorganic helicenes. However, achieving such architectures requires the introduction of poorly σ-donating and sterically rigid ligands to stabilize electron-rich metal rings, lock the helical conformation, and suppress racemization. Previous studies have shown that organic molecular cages can serve as templates for the controlled synthesis of gold and Pd nanoparticles by using their internal functional groups (*48*–*50*), indicating that the ligand environment can guide the formation of metal nanoparticles with desired architecture. Furthermore, the reported thiolate/Ph_3_P system is inclined to favor coplanar geometry (*47*, *51*) because of the smaller size and poorer rigidity of Ph_3_P compared to triphenylantimony(III) (Ph_3_Sb), indicating that Ph_3_Sb with a poor σ-donating ability can be beneficial to the high-nuclearity and noncoplanar structures. Guided by these principles, we have synthesized a μ_6_-stibine/thiolate–protected metallic helicene, Pd_11_(PhSb)_2_(AdmS)_10_ (denoted as **Pd**_**11**_**-C**, where AdmS = 1-adamantanethiolate; **C** stands for closed rather than carbon), also termed an antimony (Sb)–centered [3]Pd helicene cluster, using Ph_3_Sb as a heavy pnictogen ligand in combination with 1-adamantanethiol (AdmSH). The structure was unambiguously characterized by electrospray ionization mass spectrometry (ESI-MS) and single-crystal x-ray diffraction (SCXRD). The cluster features a Pd_3_ triangular core ortho-fused with two μ_6_-Sb–centered Pd_6_ rings, assembling into an unprecedented all-metal helicene framework reminiscent of a slightly open-mouthed scallop. This helical architecture can be unfolded by completely opening the two fused Pd_6_ rings, affording a chiral homolog, Pd_11_SCl(PhSb)_2_(AdmS)_11_ (denoted as **Pd**_**11**_**-O**, where **O** stands for open rather than oxygen). Furthermore, **Pd**_**11**_**-C** exhibits exceptional electrocatalytic performance in the two-electron oxygen reduction reaction (2e^−^ ORR), delivering a hydrogen peroxide (H_2_O_2_) selectivity of up to 96.1% at 0.6 V, which was further demonstrated by in situ H_2_O_2_-driven pollutant degradation. The detailed results are discussed below.

## RESULTS

### Synthesis and characterization

**Pd**_**11**_**-C** clusters were synthesized via a one-pot method (fig. S1; see Materials and Methods for details). Briefly, palladium chloride (PdCl_2_) was dissolved in an aqueous hydrochloric acid solution, followed by addition of a toluene solution of tetraoctylammonium bromide (TOAB) to facilitate phase transfer (*52*). After the removal of the aqueous phase, Ph_3_Sb was added and the mixture was stirred for 30 min. Subsequently, AdmSH and a cold aqueous solution of sodium borohydride (NaBH_4_) were introduced simultaneously. The reaction was maintained at 0°C overnight in the dark. The crude products were washed with methanol and separated by preparative thin-layer chromatography (PTLC). Single crystals suitable for x-ray diffraction were obtained by vapor diffusion of methanol into a dichloromethane solution of the purified products at −4°C, affording black block-shaped crystals after 1 week (fig. S2A). **Pd**_**11**_**-O** clusters were obtained in a similar manner, except that copper(II) trifluoroacetate hydrate was added (see Materials and Methods for details). Black block-shaped crystals were similarly obtained after 1 week (fig. S2B). Note that although the two Pd clusters cannot be obtained as a mixture, they can be readily distinguished by PTLC because of their distinct *R*_f_ (retention factor) values (0.6 for **Pd**_**11**_**-C** and 0.4 for **Pd**_**11**_**-O**). The two Pd clusters also exhibit markedly different solution and solid-state colors, which is further corroborated by their clearly distinguishable ultraviolet-visible-near-infrared (UV-Vis-NIR) absorption spectra (fig. S3).

**Fig. 1.**
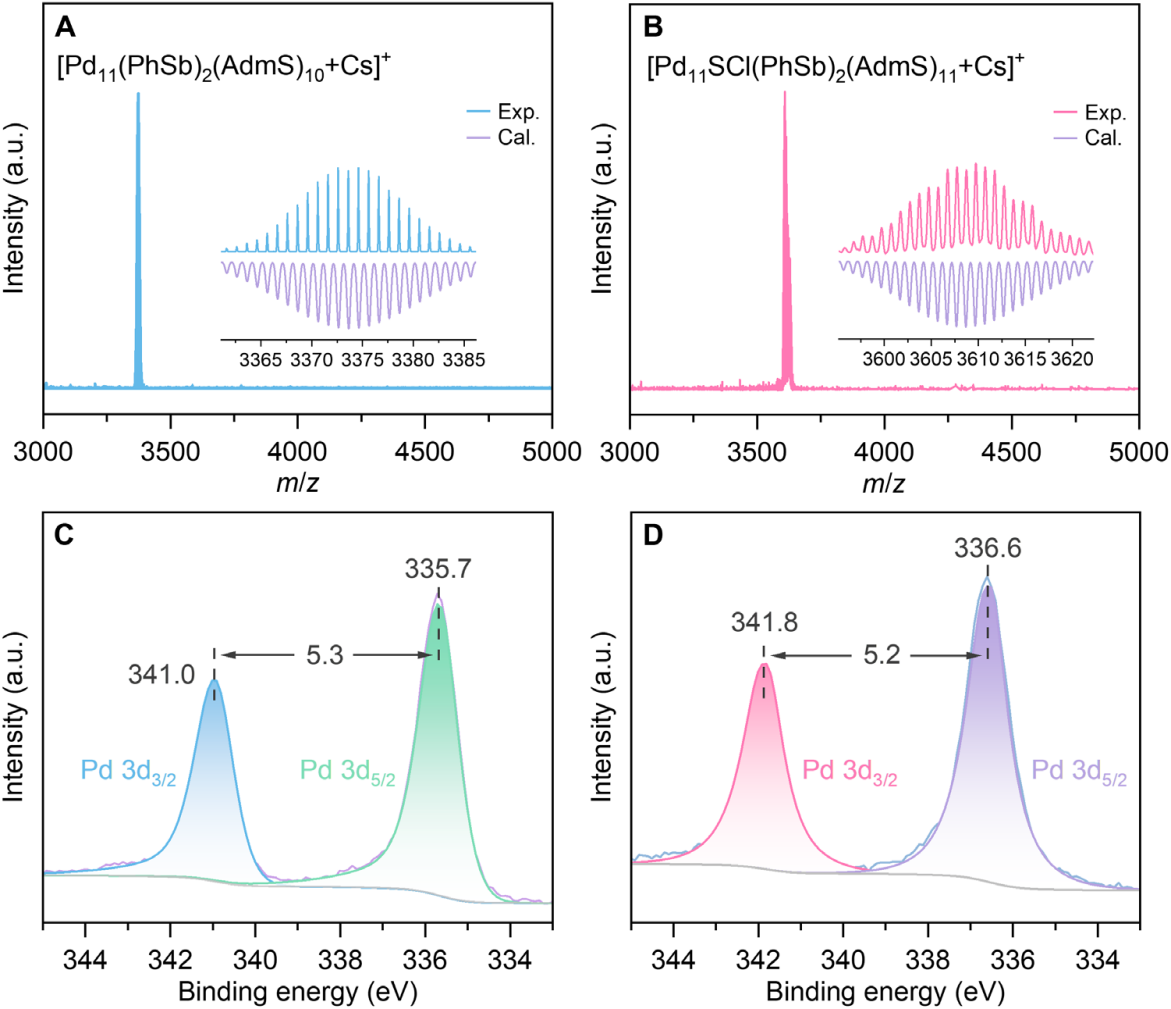
Characterization of the two Pd_11_ clusters. ESI-MS spectra of **Pd**_**11**_**-C** (**A**) and **Pd**_**11**_**-O** (**B**) clusters in positive-ion mode (the insets correspond to their isotopic patterns). XPS spectra of Pd 3d for **Pd**_**11**_**-C** (**C**) and **Pd**_**11**_**-O** (**D**) clusters. a.u., arbitrary units.

The accurate molecular compositions of the two Pd_11_ clusters were determined by ESI-MS (*53*, *54*). No detectable signal was observed in their positive or negative ion mode in the absence of Cs^+^, indicating that both clusters are electrically neutral. Upon addition of Cs^+^, the ESI-MS spectrum of **Pd**_**11**_**-C** displays a dominant peak at *m*/*z* (mass/charge ratio) 3374.11 (Fig. 1A), assigned to [Pd_11_(PhSb)_2_(AdmS)_10_Cs]^+^ (calculated: 3374.64). The well-matched isotopic distribution further corroborates this assignment (inset in Fig. 1A). Similarly, a prominent peak at *m/z* 3609.91 (Fig. 1B) in the ESI-MS of **Pd**_**11**_**-O** is readily attributed to [Pd_11_SCl(PhSb)_2_(AdmS)_11_Cs]^+^ (calculated: 3609.67; inset of Fig. 1B). X-ray photoelectron spectroscopy (XPS) and energy-dispersive x-ray spectroscopy mapping were further performed to confirm the elemental compositions and oxidation states (figs. S4 to S7). The XPS survey spectra (figs. S4A and S5A) show the expected peaks for Sb, Pd, C (carbon), and S (sulfur), as well as a faint Si (silicon) 2p peak at ∼100 eV arising from trace silica residues introduced during PTLC purification (*47*, *55*–*57*). Moreover, the high-resolution Pd 3d spectra of **Pd**_**11**_**-C** exhibit two intense peaks at 335.7 and 341.0 eV (336.6 and 341.8 eV for **Pd**_**11**_**-O**; Fig. 1, C and D), characteristic of Pd(0), indicating that the Pd atoms in both clusters are present in the zero-valent state (*58*).

**Fig. 2.**
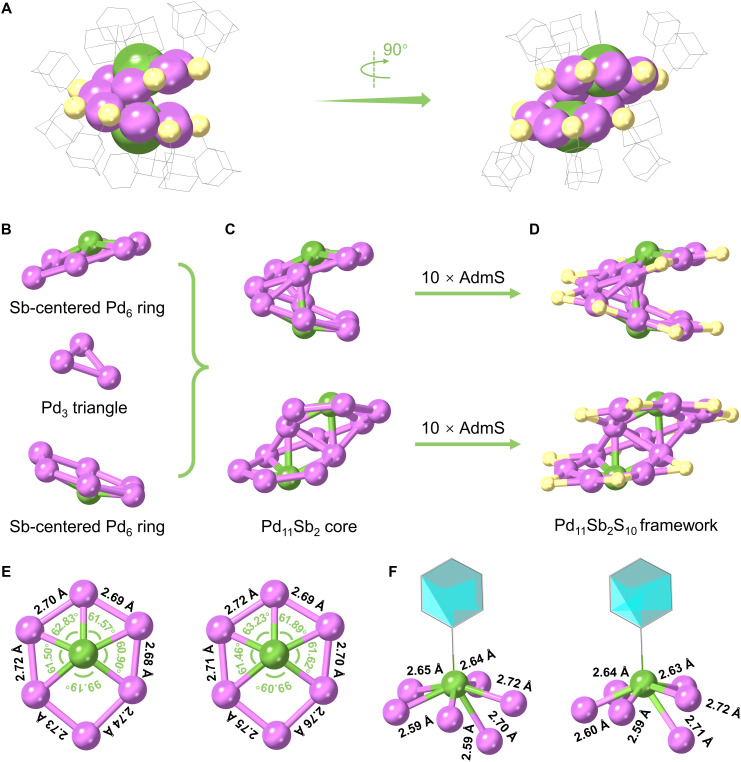
Analysis of the structure of the Pd_11_-C cluster. (**A**) Side and front views of the overall structure of the **Pd**_**11**_**-C** cluster. (**B**) Two Sb-centered Pd_6_ rings and a Pd_3_ triangle. (**C**) Pd_11_Sb_2_ core. (**D**) Framework of **Pd**_**11**_**-C** clusters by removal of all carbon and hydrogen atoms. (**E**) Two Sb-centered Pd_6_ rings. (**F**) Coordination modes of two Sb atoms and corresponding Sb─Pd bond lengths. Color codes: purple, Pd; yellow, S; green, Sb; gray, C. All hydrogen atoms are omitted for clarity.

SCXRD analysis was used to elucidate the atomically precise structures and compositions. **Pd**_**11**_**-C** crystallizes in the triclinic space group *P*-1 with two independent **Pd**_**11**_**-C** clusters per unit cell (table S1 and fig. S8). Figure 2A shows the overall structural of **Pd**_**11**_**-C** in side and front views. As illustrated in Fig. 2 (B and C), the metallic backbone of **Pd**_**11**_**-C** can be described as a Pd_3_ triangle ortho-fused to two Sb-centered Pd_6_ rings. The resulting Pd_11_Sb_2_ core is further stabilized by 10 bridging thiolate ligands, which complete the **Pd**_**11**_**-C** framework (Fig. 2D). Notably, each Sb atom from the PhSb^2−^ ligand bridges six Pd atoms (μ_6_-Sb), a coordination mode that is extremely rare in both metal cluster and pnictogen chemistry. Accordingly, **Pd**_**11**_**-C** represents an example of a hypercoordination μ_6_-stibine/thiolate–protected metallic cluster (Fig. 2A). The two Sb-centered Pd_6_ rings are structurally analogous, displaying no notable differences in Pd─Pd bond lengths or bond angles (Fig. 2E and figs. S9 and S10). The Pd─Pd distances span 2.67 to 2.84 Å with an average of 2.72 Å (fig. S9), closely matching that of bulk Pd (2.75 Å) (*59*), indicative of pronounced metallic bonding. In addition, the Sb─Pd bonds associated with the two μ_6_-Sb centers are nearly identical (Fig. 2F), with a mean Sb─Pd distance of 2.65 Å (fig. S11), comparable to that observed in the [Pd_4_(μ_3_-SbMe_3_)_4_(SbMe_3_)_4_] (*60*).

**Fig. 3.**
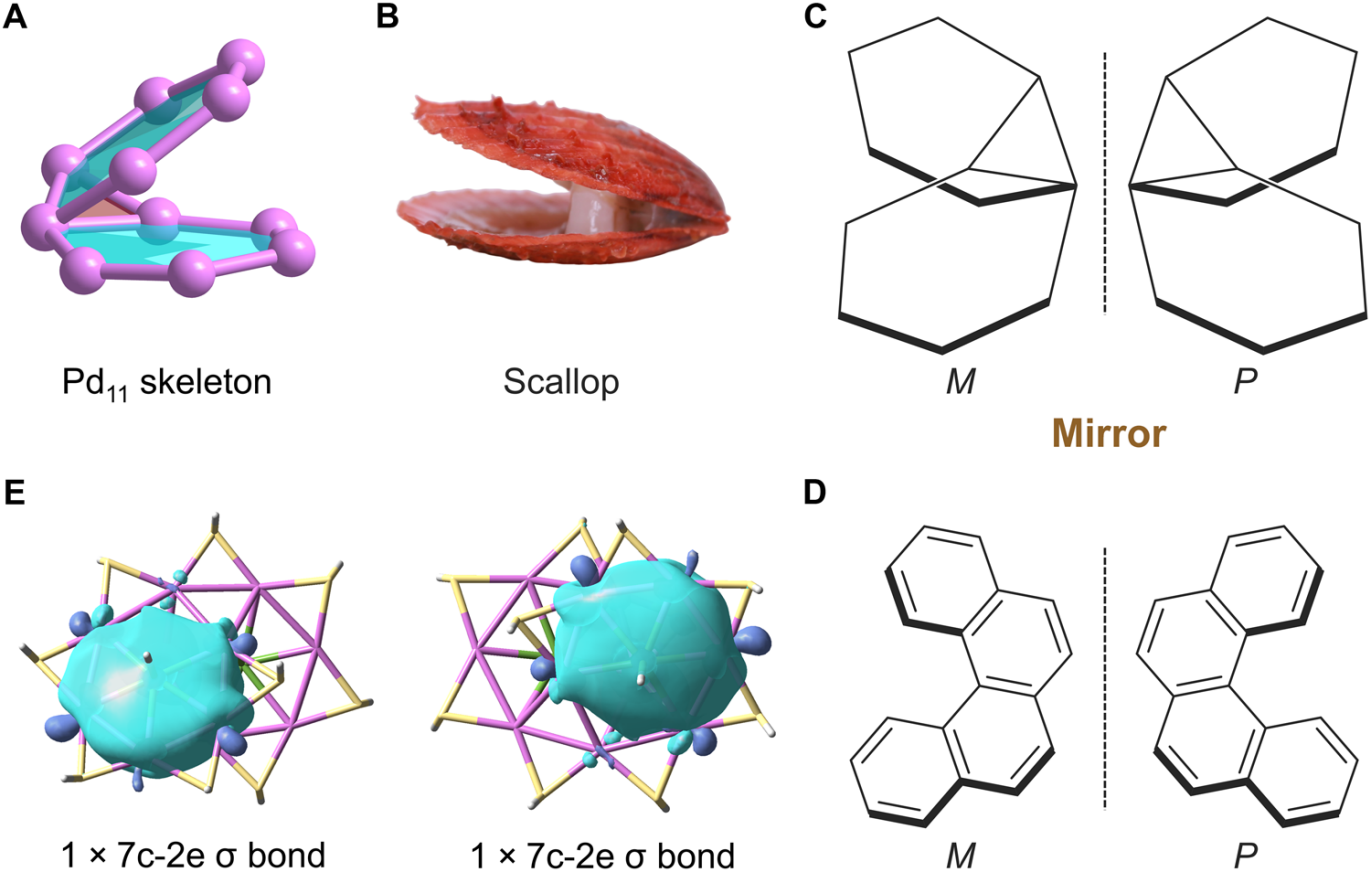
All-metal helicene structure and bonding patterns of Pd_11_-C clusters. (**A**) Scallop-like Pd_11_ skeleton. (**B**) Photograph of a slightly open-mouthed scallop. (**C**) Pair of [3]Pd-helicene enantiomers. (**D**) Pair of [4]carbohelicene enantiomers. (**E**) Bonding patterns of Sb-centered Pd_6_ rings from adaptive natural density partitioning analyses. Color codes: purple, Pd; yellow, S; green, Sb; white, H.

Notably, the Pd_11_ skeleton resembles a slightly open-mouthed scallop (Fig. 3, A and B) and exhibits a twisted helical configuration arising from the ortho-fusion of a triangular Pd_3_ plane with two Pd_6_ planes (fig. S12), thereby giving rise to a helically chiral **Pd**_**11**_**-C** cluster. The dihedral angles between three fused rings are 34.59°, 49.00°, and 21.19° (fig. S12). As expected, a pair of enantiomers is present in the unit cell of **Pd**_**11**_**-C** (fig. S8). To visualize the origin of helicity, the key metallic backbones responsible for chirality were extracted and highlighted (Fig. 3C), which are structurally analogous to the classical *M*- and *P*-helicenes, exemplified by [4]carbohelicene (Fig. 3D). Accordingly, the **Pd**_**11**_**-C** clusters can be named as [3]Pd-helicene on the basis of the established helicene nomenclature (*61*, *62*), to the best of our knowledge, a previously unknown metallic helicene molecule with an all-metal helical backbone.

From a theoretical standpoint, such a metallic helicene is expected to be thermodynamically fragile because of the intrinsic conflict between the nondirectional nature of metallic bonding and the stringent geometric constraints imposed by a helical topology. Adaptive natural density partitioning analysis reveals the presence of two delocalized 7c-2e σ bonds within the Pd_11_ core (Fig. 3E), each localized on an Sb-centered Pd_6_ ring, which plays a crucial role in consolidating the all-metal helical framework. In addition, intramolecular C─H···π interactions between the adamantyl and phenyl moieties further contribute to the stabilization of this ligand-protected metallic helicene (fig. S13).

**Fig. 4.**
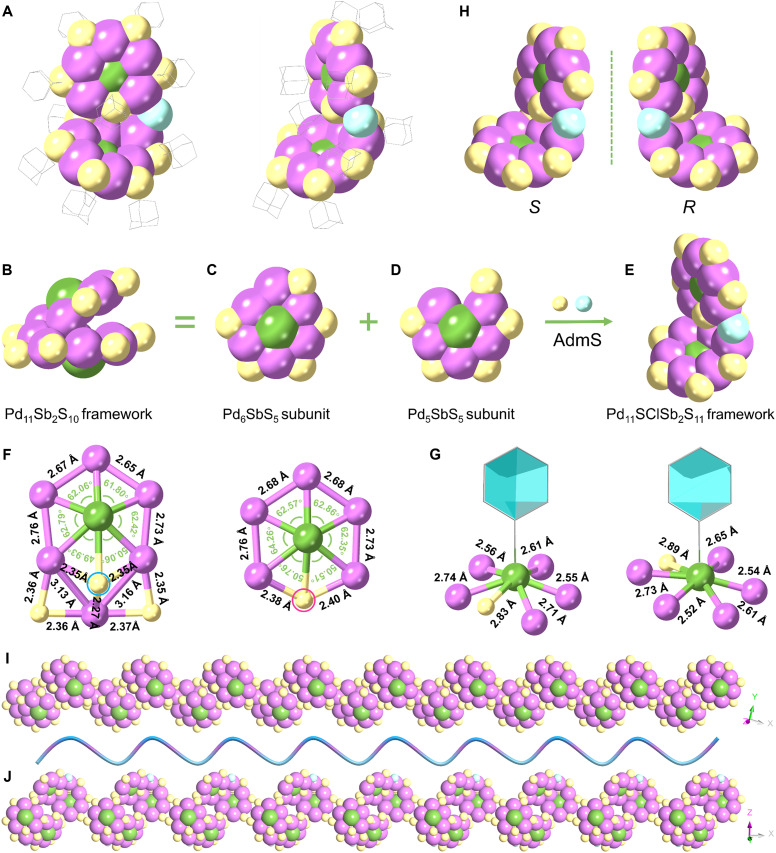
Structural evolution and self-assembly of the two Pd_11_ clusters. (**A**) Total structure of the **Pd**_**11**_**-O** cluster. (**B** to **E**) Conceptually structural evolution from the **Pd**_**11**_**-C** to **Pd**_**11**_**-O** cluster. (**F** and **G**) Substructure units of the **Pd**_**11**_**-O** cluster. (**H**) Pair of enantiomers for **Pd**_**11**_**-O** clusters. (**I**) Self-assembled *M*- and *P*-**Pd**_**11**_**-C** clusters pack into zigzag arrangements of opposite-handed segments. (**J**) Self-assembled *R*- and *S*-**Pd**_**11**_**-O** clusters pack into zigzag arrangements of opposite-handed segments. Color codes: purple, Pd; yellow, S; green, Sb; blue, Cl; gray, C. All hydrogen atoms are omitted for clarity.

The metallic helicene framework of **Pd**_**11**_**-C** can be disrupted by subtle variations in the reaction conditions, affording a homolog, **Pd**_**11**_**-O** (Fig. 4A), which adopts a fully open scallop-like architecture. To elucidate the structural relationship between the two Pd_11_ clusters, a conceptual rather than chemical transformation pathway from **Pd**_**11**_**-C** to **Pd**_**11**_**-O** is dissected in Fig. 4 (B to E) and fig. S14 (A to D). The process is initiated by cleavage at the shared Pd site (highlighted in orange in fig. S14A), leading to the generation of Pd_6_SbS_5_ and Pd_5_SbS_5_ subunits through successive breaking of Pd─Pd, Sb─Pd, and Pd─S bonds (Fig. 4, C and D, and fig. S14, B and C). Subsequently, these two subunits are reconnected via a chlorine bridge and a bridging μ_3_-sulfur (highlighted with a green circle in fig. S14D), accompanied by a slight migration of a preexisting S atom [red circle in fig. S14 (C and D)] toward a neighboring Pd atom. The final **Pd**_**11**_**-O** structure is completed by insertion of an additional S atom (blue circle) and a bridging AdmS^−^ ligand (purple circle), culminating in the fully opened **Pd**_**11**_**-O** cluster (Fig. 4, A and E, and fig. S14D). Despite this pronounced structural rearrangement, the Pd─Pd and Sb─Pd bond lengths and associated angles remain largely unchanged (Fig. 4, F and G, and figs. S15 to S17). The dominant structural difference is the formation of two additional Sb─S bonds, involving one μ_4_-S atom (cyan circle) and one μ_3_-S atom (pink circle), as shown in Fig. 4F. A detailed comparison of bond metrics for **Pd**_**11**_**-C** and **Pd**_**11**_**-O** is provided in figs. S18 and S19. In addition, the intramolecular C─H···Cl (fig. S20) and C─H···π interactions are present in **Pd**_**11**_**-O** (fig. S21).

Although **Pd**_**11**_**-O** lacks the spiral helicity characteristic of **Pd**_**11**_**-C**, it remains chiral, with a pair of enantiomers coexisting in the unit cell (Fig. 4H and fig. S22). In well-defined metal clusters, chirality commonly originates from an asymmetry metal core, the arrangement of staple motifs, or chiral ligand (*63*–*66*). Here, both the distorted Pd cores and the asymmetric organization of staple motifs in Pd_6_SClSbS_6_ and Pd_5_SbS_5_ subunits are responsible for the chirality of **Pd**_**11**_**-O** (fig. S23). Similar to **Pd**_**11**_**-C**, **Pd**_**11**_**-O** further packs into zigzag arrangements of opposite-handed segments in the crystal lattice through intermolecular H···H interactions (Fig. 4, I and J, and figs. S24 and S25). The packing motifs of **Pd**_**11**_**-C** and **Pd**_**11**_**-O** within 3 × 3 × 3 unit cells are also presented in figs. S26 and S27.

**Fig. 5.**
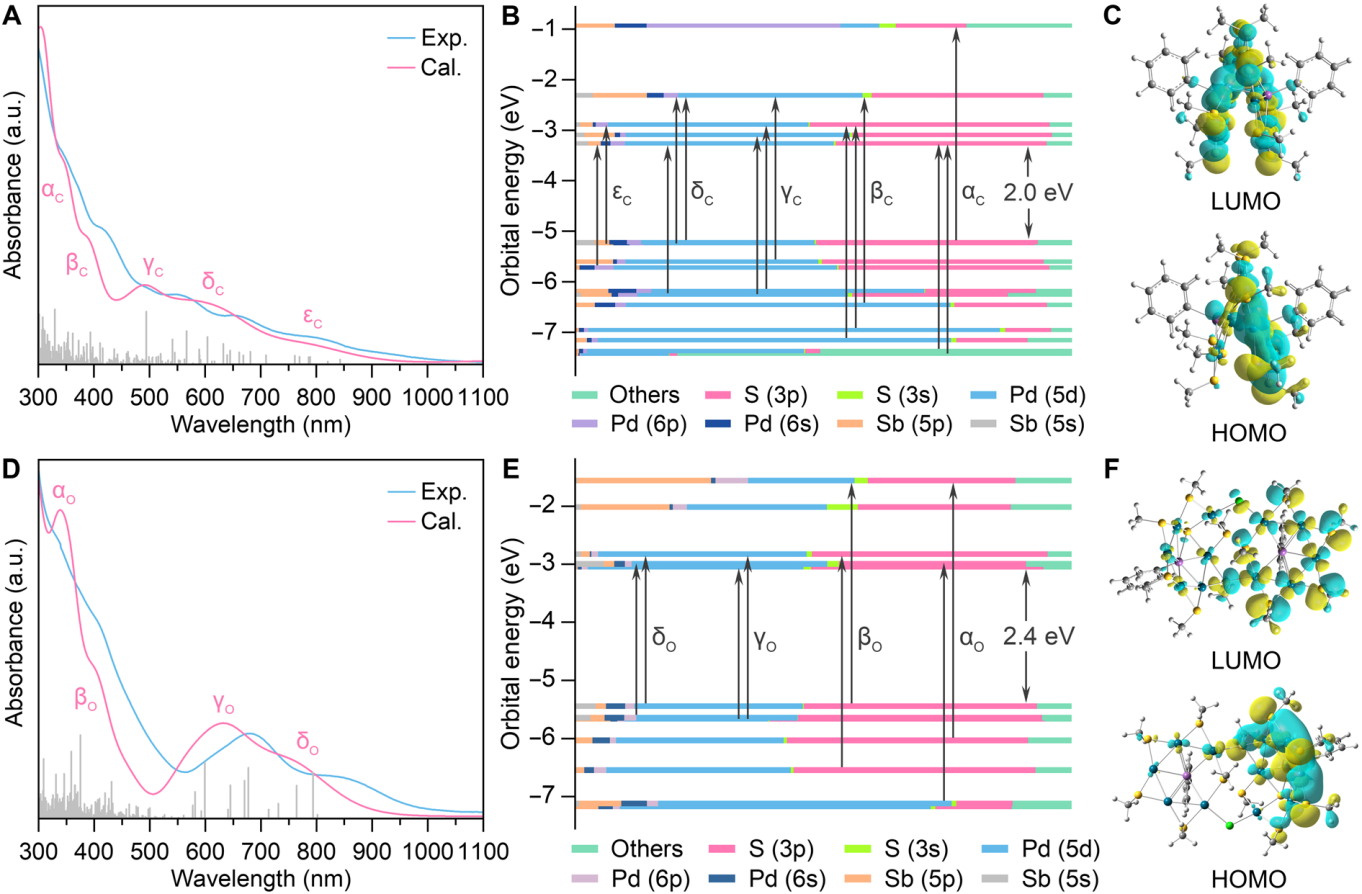
Optical absorption properties and electronic structures of the Pd_11_ clusters. Experimental and calculated UV-Vis-NIR spectra of **Pd**_**11**_**-C** (**A**) and **Pd**_**11**_**-O** (**D**) clusters. Kohn-Sham molecular orbital energy diagrams of **Pd**_**11**_**-C** (**B**) and **Pd**_**11**_**-O** (**E**) clusters. HOMO and LUMO distributions of **Pd**_**11**_**-C** (**C**) and **Pd**_**11**_**-O** (**F**) clusters.

Time-dependent density functional theory (DFT) calculations were performed to elucidate the electronic structures and absorption properties of the two Pd_11_ clusters. As shown in Fig. 5A, the simulated absorption spectrum of **Pd**_**11**_**-C** is in good agreement with the experimental data. The five major absorption bands at 353, 393, 494, 604, and 789 nm were assigned on the basis of Kohn-Sham molecular orbital analysis (Fig. 5B). The absorption peak at 353 nm is mainly dominated by the transitions of HOMO (highest occupied molecular orbital) → LUMO+8, HOMO−30 → LUMO (lowest unoccupied molecular orbital), and HOMO−31 → LUMO (fig. S28). Notably, the electron densities of HOMO−30 and HOMO−31 are mainly localized on the phenyl ring, indicating the coexistence of metal-to-metal charge transfer and ligand-to-metal charge transfer. In contrast, the remaining absorption features are predominantly associated with transitions involving Pd 5d and S 3p orbitals (Fig. 5, B and C, and fig. S28), confirming that the thiolate-stabilized Sb-centered [3]Pd-helicene framework makes the principal contribution to the optical response. **Pd**_**11**_**-O** exhibits markedly different absorption characteristics, which are well reproduced by the time-dependent DFT calculations (Fig. 5D). The absorption peak at 334 nm is attributed to the transitions of HOMO−3 → LUMO+7 and HOMO−20 → LUMO+1, with negligible contributions from the phenyl or adamantyl moieties (Fig. 5E and fig. S29). Both the HOMOs and LUMOs of **Pd**_**11**_**-O** are largely localized on one of the two fused subunits, primarily involving Pd 5d and S 3p orbitals, which also dominate the absorptions at 334, 409, 636, and 758 nm (Fig. 5, E and F, and fig. S29). Correspondingly, the electron density in **Pd**_**11**_**-O** is distributed over the individual Pd_6_SClSbS_6_ and Pd_5_SbS_5_ rings rather than delocalized over a continuous all-metal helical framework, as shown in **Pd**_**11**_**-C**. This more fragmented electronic structure likely accounts for the comparatively inert nature of **Pd**_**11**_**-O** (*67*). Consistent with this interpretation, **Pd**_**11**_**-O** exhibits a larger HOMO-LUMO energy gap and higher thermal stability than **Pd**_**11**_**-C** (Fig. 5, B and E, and fig. S30).

### Electrocatalytic ORR performance and practical applications

**Fig. 6.**
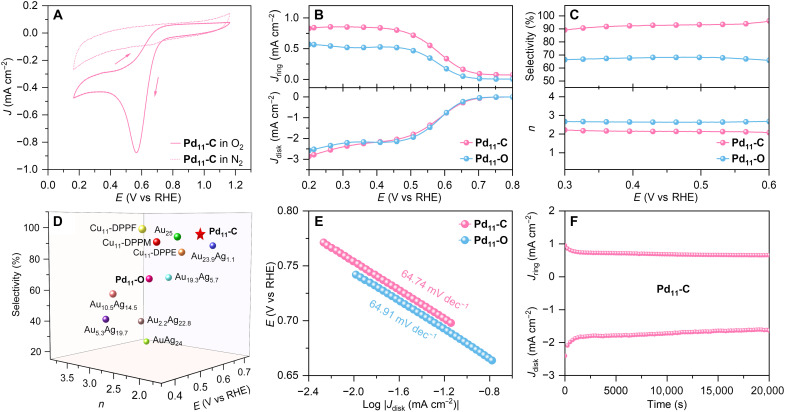
Electrocatalytic performance toward 2e^−^ ORR in the alkaline electrolyte. (**A**) CV curves of **Pd**_**11**_**-C** clusters in O_2_-saturated and N_2_-saturated 0.1 M KOH. (**B**) LSV curves of Pd_11_ clusters obtained at 1600 rpm and 10 mV s^−1^ in O_2_-saturated 0.1 M KOH. (**C**) H_2_O_2_ selectivity and electron transfer number based on LSV data of Pd_11_ clusters. (**D**) Performance comparison of H_2_O_2_ selectivity among cluster materials in the ORR. (**E**) Tafel slopes of Pd_11_ clusters. (**F**) Stability test of **Pd**_**11**_**-C** clusters at 0.5 V (versus RHE) in 0.1 M KOH for 20,000 s.

Motivated by the foregoing analyses, we anticipated that **Pd**_**11**_**-C** would exhibit higher electrocatalytic activity than its opened counterpart **Pd**_**11**_**-O**, which was verified by ORR electrocatalysis measurements. The electrocatalytic ORR performance was evaluated in oxygen gas (O_2_)–saturated 0.1 M potassium hydroxide (KOH) using a rotating ring-disk electrode (*68*). Before testing, the collection efficiency of the rotating ring-disk electrode was calibrated in a mixed electrolyte containing 5 mM potassium ferricyanide {K_3_[Fe(CN)_6_]} and 0.1 M KOH, yielding a value of 0.362 (fig. S31), in good agreement with the theoretical value of 0.37. Cyclic voltammetry (CV) profiles of **Pd**_**11**_**-C** clusters were recorded in O_2_-saturated and nitrogen gas (N_2_)–saturated 0.1 M KOH at 50 mV s^−1^ (Fig. 6A). A pronounced cathodic peak at 0.57 V is observed only in the presence of O_2_ and disappears under N_2_, confirming its ORR activity. **Pd**_**11**_**-O** exhibits similar behavior (fig. S32). Linear sweep voltammetry (LSV) curves were recorded at 1600 rpm in O_2_-saturated 0.1 M KOH to probe the ORR selectivity, with the disk electrode detecting O_2_ reduction and the ring electrode monitoring H_2_O_2_ oxidation (*69*). As shown in Fig. 6B, **Pd**_**11**_**-C** delivers a substantially higher ring current density than **Pd**_**11**_**-O** over the potential range of 0.3 to 0.6 V [versus reversible hydrogen electrode (RHE)], suggesting a stronger preference for the 2e^−^ ORR pathway.

The calculated H_2_O_2_ selectivity and the electron transfer number (*n*) are plotted in Fig. 6C. Within 0.3 to 0.6 V (versus RHE), **Pd**_**11**_**-C** exhibits a H_2_O_2_ selectivity exceeding 89.3%, with a maximum of 96.1% at 0.6 V, notably higher than that of **Pd**_**11**_**-O**, with corresponding *n* values ranging from 2.07 to 2.21, confirming the dominance of the 2e^−^ ORR route. Figure 6D shows the H_2_O_2_ selectivity of the two Pd_11_ and previously reported cluster catalysts (*70*, *71*). The ORR Tafel slope of **Pd**_**11**_**-C** (64.74 mV dec^−1^) is also slightly lower than that of **Pd**_**11**_**-O** (64.91 mV dec^−1^) (Fig. 6E). To exclude surface area effects, the electrochemically active surface area was estimated from the double-layer capacitance (*C*_dl_) extracted from nonfaradaic CV measurements (fig. S33). The comparable *C*_dl_ values for **Pd**_**11**_**-C** (0.3001 mF cm^−2^) and **Pd**_**11**_**-O** (0.3232 mF cm^−2^) (fig. S34) suggest that the electrochemically active surface area is not responsible for their different performances. Electrochemical impedance spectroscopy reveals that **Pd**_**11**_**-O** shows a lower charge-transfer resistance than **Pd**_**11**_**-C** (fig. S35) despite its inferior ORR activity, indicating that charge-transfer kinetics are not the determining factor. Last, the durability of **Pd**_**11**_**-C** was evaluated by chronoamperometry at fixed potentials (0.5 V at the disk and 1.3 V at the ring versus RHE). As shown in Fig. 6F, **Pd**_**11**_**-C** exhibits stable activity and H_2_O_2_ selectivity over 20,000 s, demonstrating its superior electrocatalytic stability.

**Fig. 7.**
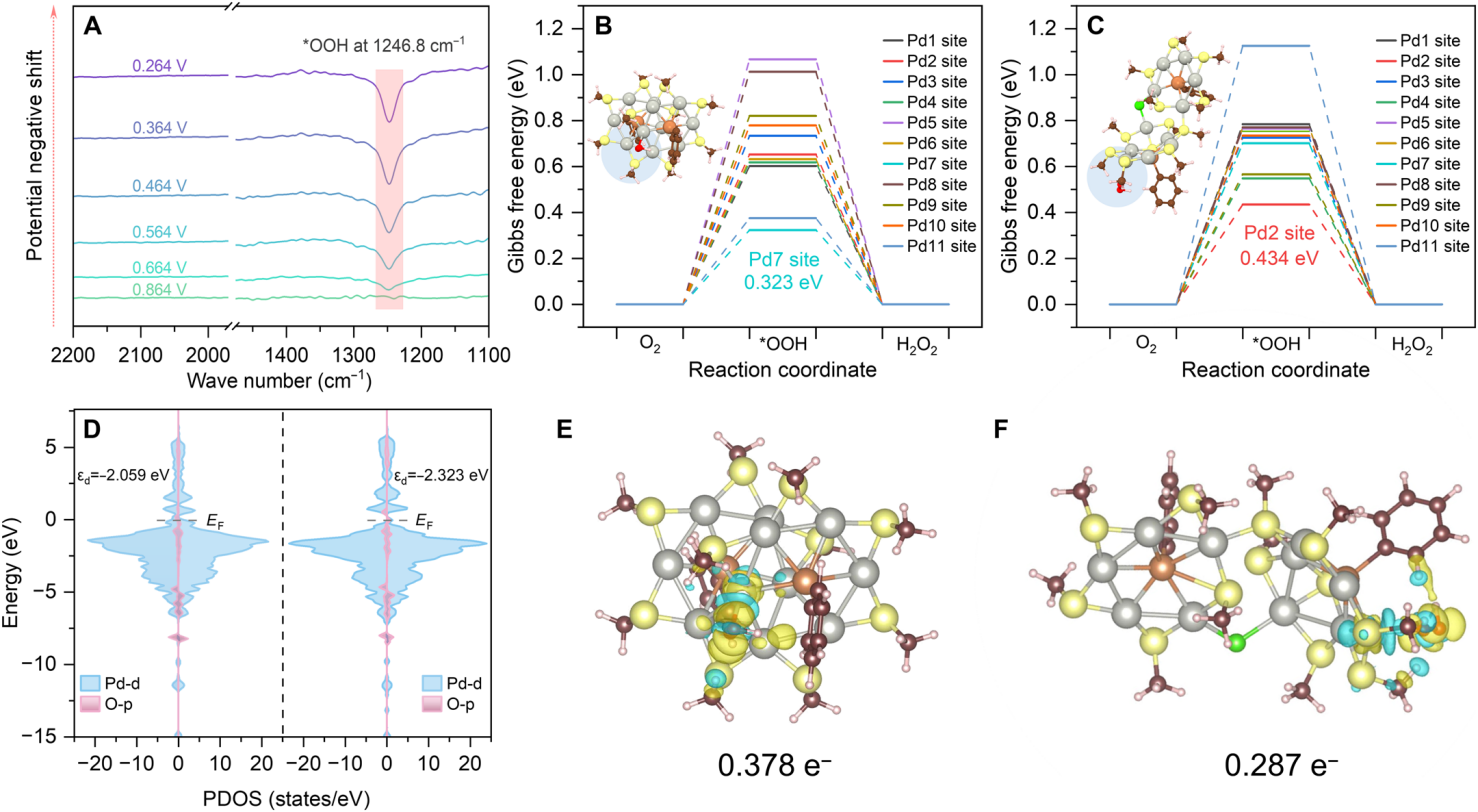
Operando infrared spectroscopy and theoretical calculations. (**A**) Potential-dependent operando ATR-SEIRAS spectra of **Pd**_**11**_**-C** clusters in O_2_-saturated 0.1 M KOH electrolyte at different potentials from 0.864 to 0.264 V (versus RHE). Free energy diagrams of the rate-determining step on **Pd**_**11**_**-C** (**B**) and **Pd**_**11**_**-O** (**C**) clusters. (**D**) Projected density of states (PDOS) of *OOH adsorbed on **Pd**_**11**_**-C** (left) and **Pd**_**11**_**-O** (right) clusters. The charge density difference mappings between *OOH and the optimal active site on **Pd**_**11**_**-C** (**E**) and **Pd**_**11**_**-O** (**F**) clusters.

Operando attenuated total reflectance surface-enhanced infrared absorption spectroscopy (ATR-SEIRAS) was performed to monitor the adsorbed intermediates on **Pd**_**11**_**-C** during ORR. As shown in Fig. 7A, a distinct peak at 1246.8 cm^−1^ appears, corresponding to the O─O stretching vibration of the *OOH intermediate. Its intensity grows steadily as the potential shifts from 0.864 to 0.264 V (versus RHE), evidencing the accumulation of *OOH species and 2e^−^ ORR pathway. DFT calculations were implemented to elucidate the origin of the difference in 2e^−^ ORR performances of **Pd**_**11**_**-C** and **Pd**_**11**_**-O**. The well-defined structural models were constructed from the crystallographic configurations of the two Pd_11_ clusters. All Pd sites on **Pd**_**11**_**-C** and **Pd**_**11**_**-O** clusters serve as potential catalytic active sites (figs. S36 and S37). As illustrated in Fig. 7 (B and C), the O_2_ → *OOH step is identified as the rate-determining step for both **Pd**_**11**_**-C** and **Pd**_**11**_**-O**, indicating that an enhanced *OOH adsorption is beneficial to 2e^−^ ORR (*72*, *73*). **Pd**_**11**_**-C** achieves its optimal 2e^−^ ORR performance at the Pd7 site, with an energy barrier of 0.323 eV, whereas the Pd5 site shows a higher barrier of 1.067 eV, corresponding to weaker adsorption (Fig. 7B). For **Pd**_**11**_**-O**, the Pd2 site has the lowest energy barrier of 0.434 eV, with its Pd11 site displaying the highest energy barrier of 1.126 eV, implying the weakest adsorption (Fig. 7C). Projected density of states (PDOS) analysis (Fig. 7D) reveals that **Pd**_**11**_**-C** exhibits an upshifted d-band center of −2.059 eV in comparison with that of **Pd**_**11**_**-O** of −2.323 eV, thereby strengthening *OOH adsorption (*74*). Concordantly, the Pd-d orbitals of **Pd**_**11**_**-C** highly overlap with the O-p orbitals of the *OOH intermediate near the Fermi level (*E*_F_), indicating their stronger orbital hybridization. Differential charge density mappings (Fig. 7, E and F) also indicate a higher charge transfer (0.378 e^−^) between the Pd7 site and *OOH in **Pd**_**11**_**-C** compared to that of **Pd**_**11**_**-O** (0.287 e^−^), further confirming the strong *OOH adsorption of the Pd7 site. Collectively, these results demonstrate that the superior electrocatalytic oxygen reduction performance of **Pd**_**11**_**-C** could originate from its upshifted d-band center, which lowers the rate-determining energy barrier of O_2_ → *OOH and promotes the 2e^−^ ORR pathway.

**Fig. 8.**
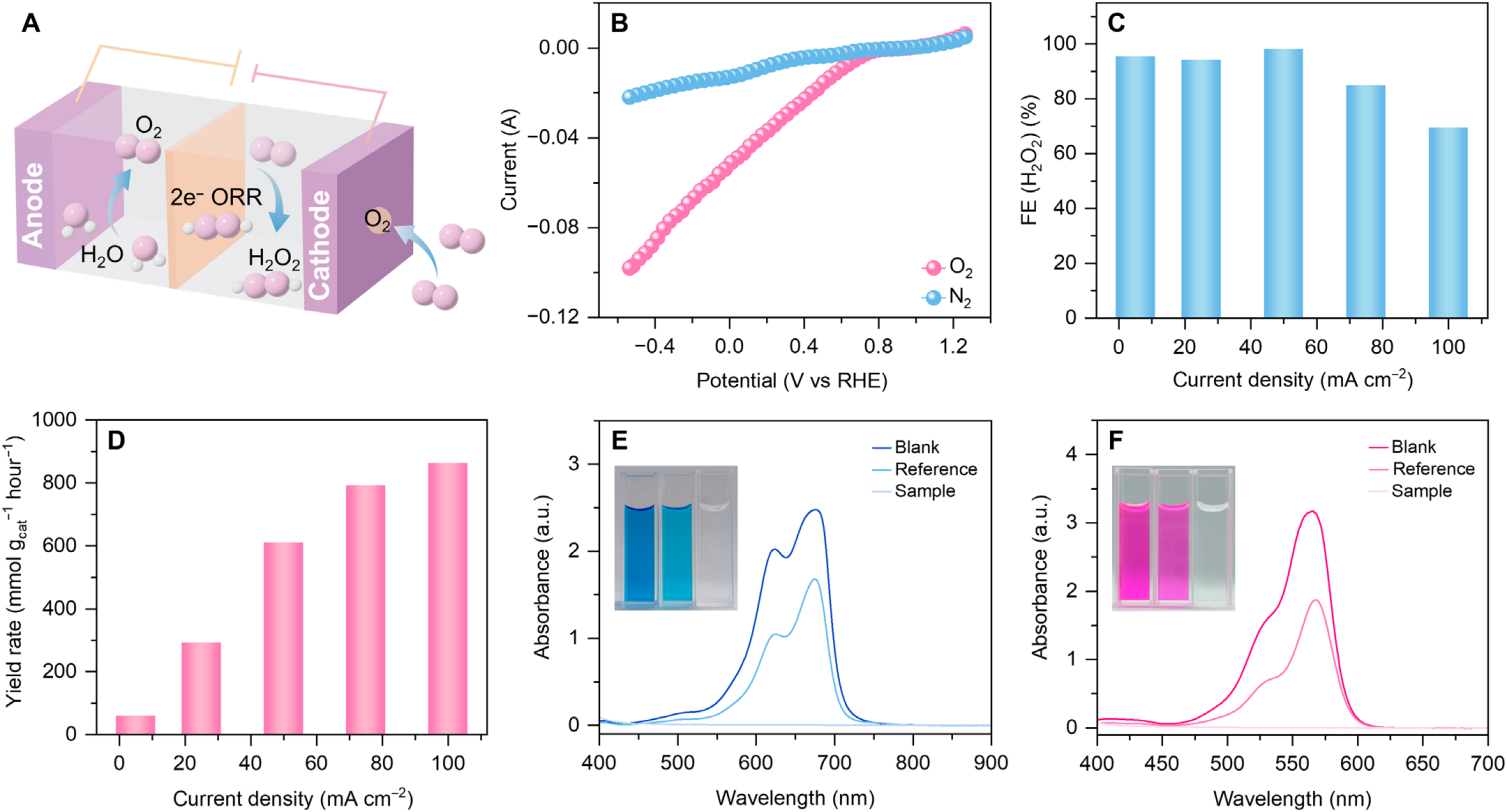
In situ H_2_O_2_ generation in flow cells and related applications. (**A**) Schematic diagram of the flow cell device for H_2_O_2_ electrosynthesis. (**B**) LSV curves of **Pd**_**11**_**-C** clusters in an O_2_/N_2_–saturated KOH electrolyte in the flow cell. (**C**) FE(H_2_O_2_) of **Pd**_**11**_**-C** clusters at different current densities. (**D**) H_2_O_2_ yield rate of **Pd**_**11**_**-C** clusters at different current densities. Digital images and corresponding UV-Vis absorption spectra of MB (**E**) and RhB (**F**) solutions after the addition of the prepared Fenton’s reagent with different catholytes.

To evaluate the practical applicability of **Pd**_**11**_**-C** for H_2_O_2_ electrosynthesis, its performance was further examined in a three-phase flow cell (Fig. 8A). The cathode was fabricated by coating **Pd**_**11**_**-C** onto a gas-diffusion electrode. As presented in Fig. 8B, the LSV recorded under O_2_ flow exhibits a pronounced current increase at more negative potentials, whereas the response is strongly suppressed under N_2_, confirming that the observed activity originates from the ORR. The H_2_O_2_ production performance was assessed by galvanostatic electrolysis over a wide range of current densities (fig. S38). The concentration of generated H_2_O_2_ was quantified using a calibration curve obtained by correlating the absorbance at 316 nm with the concentration of cerium(IV) sulfate [Ce(SO_4_)_2_] on the basis of the UV-Vis absorption spectra of standard solutions (fig. S39). The faradaic efficiency (FE) for H_2_O_2_ production remains between 98.1 and 69.4% across current densities from 5 to 100 mA cm^−2^ (Fig. 8C). Meanwhile, the H_2_O_2_ production rate increases sharply with the current density (Fig. 8D), reaching 863.3 mmol g_cat_^−1^ hour^−1^ at 100 mA cm^−2^. The durability of the **Pd**_**11**_**-C**–based cathode was further evaluated at 25 mA cm^−2^ (fig. S40), during which the potential and FE(H_2_O_2_) remained stable (>89.1%), demonstrating excellent long-term stability. Moreover, UV-Vis-NIR absorption spectra and ESI-MS analyses indicated that the molecular structure and composition of the **Pd**_**11**_**-C** catalyst remained intact after stability testing (figs. S41 and S42). The practical utility of the in situ–generated H_2_O_2_ was demonstrated through the Fenton degradation of organic pollutants. Methylene blue (MB) and rhodamine B (RhB) were selected as model contaminants. The catholyte obtained at 25 mA cm^−2^ was used as the H_2_O_2_ source, and the Fenton reaction was initiated by adding Fe^2+^ (2 mM) and adjusting the pH to ∼3 with H_2_SO_4_ (sulfuric acid). After brief shaking, the solutions became colorless and transparent (Fig. 8, E and F). UV-Vis analysis confirmed efficient degradation of both MB and RhB, attributable to the generation of hydroxyl radicals (•OH). The pivotal role of •OH was verified by scavenger experiments: The addition of *tert*-butanol markedly inhibited dye degradation (fig. S43) (*75*, *76*). Collectively, these results highlight the excellent selectivity, stability, and practical potential of the **Pd**_**11**_**-C** catalyst in H_2_O_2_ electrosynthesis and wastewater remediation.

## DISCUSSION

In summary, we have successfully synthesized and atomically characterized a [3]Pd helicene cluster (**Pd**_**11**_**-C**) by ESI-MS, SCXRD, and complementary techniques. This cluster adopts a scallop-shaped all-metal Pd_11_ helical geometry composed of a central Pd_3_ triangle fused with two Pd_6_ rings, which are further stabilized by two 7c-2e σ bonds. Cleavage of the fused Pd_6_ rings converts the metallic helicene into a chiral opened homolog, **Pd**_**11**_**-O**. Electronic structure analyses reveal that the all-metal helicene skeleton has high electron density, endowing **Pd**_**11**_**-C** with outstanding catalytic activity toward the 2e^−^ ORR, delivering a H_2_O_2_ selectivity of up to 96.1% at 0.6 V, a FE(H_2_O_2_) reaching 98.1% at 50 mA cm^−2^, and a production rate of 863.3 mmol g_cat_^−1^ hour^−1^ at 100 mA cm^−2^, thereby enabling efficient in situ H_2_O_2_ generation for pollutant degradation. Operando ATR-SEIRAS analysis combined with theoretical calculations indicates that the upshifted d-band center lowers the rate-determining energy barrier of O_2_ → *OOH, thereby effectively promoting the 2e^−^ ORR pathway toward H_2_O_2_. This work represents an important advance in pursuit of all-metal helicene and opens avenues for the rational synthesis and functional exploitation of larger helicene clusters with an all-metal skeleton.

## MATERIALS AND METHODS

PdCl_2_, AdmSH, copper(II) trifluoroacetate hydrate [Cu(CF_3_COO)_2_·*x*H_2_O], TOAB, Ph_3_Sb, NaBH_4_, carbon powder, Ce(SO_4_)_2_, KOH, K_3_[Fe(CN)_6_], *tert*-butanol, MB, RhB, toluene, methanol, ethanol, dichloromethane, petroleum ether, hydrochloric acid, and H_2_SO_4_ were all purchased from Sinopharm Chemical Reagent Co., Ltd. Nafion solution (5 wt %) was purchased from Shanghai Hesen Electrical Co., Ltd. All reagents were used as received without further purification. Ultrapure water was used in all experiments.

### Synthesis of Pd_11_ clusters

In this work, the two Pd_11_ clusters were synthesized using a simple liquid-phase route. Typically, taking the synthesis of **Pd**_**11**_**-C** clusters as an example, PdCl_2_ (10.00 mg, 0.056 mmol) was dissolved in an aqueous hydrochloric acid solution, followed by addition of a toluene solution of TOAB (46.00 mg, 0.084 mmol) to facilitate phase transfer. After the removal of the aqueous layer, Ph_3_Sb (39.54 mg, 0.112 mmol) was added, and the mixture was stirred for 30 min. Subsequently, AdmSH (37.69 mg, 0.224 mmol) and a cold aqueous solution of NaBH_4_ (15.89 mg, 0.420 mmol) were introduced simultaneously, resulting in an immediate color change to dark brown. The reaction was maintained at 0°C overnight in the dark. The crude products were washed with methanol and separated by PTLC. A mixture of dichloromethane and petroleum ether was used as the developing solvent with a volume ratio of 1:2. Single crystals suitable for x-ray diffraction were obtained by vapor diffusion of methanol into a dichloromethane solution of the purified products at −4°C, affording black block-shaped crystals after 1 week. **Pd**_**11**_**-O** clusters were obtained in a similar manner, except that copper(II) trifluoroacetate hydrate (16.36 mg, 0.056 mmol) was added into the solution of Pd after phase transfer.

### Characterization of Pd_11_ clusters

UV-Vis-NIR absorption spectra were recorded using a Metash UV-6100 spectrophotometer at room temperature with the samples dissolved in dichloromethane. Mass spectra were conducted on a Waters XEVO G2-XS QT mass spectrometer, and cesium acetate was added to charge the clusters. The purified samples were dissolved in toluene and electrosprayed for mass spectrometric analysis. SCXRD data were obtained using a Bruker D8 VENTURE CMOS PHOTON 100 diffractometer with a Helios MX multilayer monochromator and Cu Kα radiation. The structure was solved using Olex2 and the SHELXT program by intrinsic phasing and refined using the SHELXL package by least-squares minimization. XPS data were recorded on a Shimadzu AXIS SUPRA spectrometer equipped with a monochromatic Al Kα x-ray source. All binding energies were referenced to the C 1s peak at 284.8 eV. Scanning electron microscopy was performed on a SUPRA55 field-emission microscope at an accelerating voltage of 10 kV.

## Supplementary Materials

### This PDF file includes:

Supplementary Text Figs. S1 to S43 Tables S1 and S2 References
