# Supplementary material for: Biomimetic all-metal Pd11 helicene
Source: Sci Adv. 2026 Jul 15;12(29):eaef7488. doi: 10.1126/sciadv.aef7488 (PMC13371903; doi:10.1126/sciadv.aef7488)
Supplement: Supplementary file 1 — Supplementary Text Figs. S1 to S43 Tables S1 and S2 References [file sciadv.aef7488_sm.pdf]

Science Advances
AAAS

# Supplementary Materials for

## Biomimetic all-metal $Pd_{11}$ helicene

Yan Sun *et al.*

Corresponding author: Zibao Gan, ganzibao@lyu.edu.cn; Xiuwen Zheng, zhengxiuwen@lyu.edu.cn;
Di Sun, dsun@sdu.edu.cn

*Sci. Adv.* **12**, eaef7488 (2026)
DOI: 10.1126/sciadv.aef7488

**This PDF file includes:**

Supplementary Text
Figs. S1 to S43
Tables S1 and S2
References

## Supplementary Text

### Electronic structure calculations

All calculations were performed with the Gaussian 16 program (Revision B01) (*77*). All quantum chemistry calculations were carried out by DFT-PBE0 method (*78–80*), adding the D3 version of Grimme's dispersion with Becke-Johnson damping function (*81*). The double-zeta Turbomole series basis set, Def2-SVP, was used for all atoms (*82*). The absorption spectrum curve was generated by the Multiwfn (version 3.8 (dev)) program (*83*) from the TDDFT results. The molecular orbital composition analysis was performed by the Natural Atomic Orbital (NAO) method (*84*, *85*). For saving the computational cost, the 1-adamantane groups in $Pd_{11}(PhSb)_2(AdmS)_{10}$ and $Pd_{11}SCl(PhSb)_2(AdmS)_{11}$ molecules were simplified to $CH_3$ groups in all calculations. After simplification, the replacing H atoms were optimized, with other atoms being kept as in the crystal structure.

### Adaptive natural density partitioning (AdNDP) analysis

AdNDP (*86*) was performed using the Multiwfn program (version 3.8 (dev)) (*83*, *87*), for which the 1-adamantane groups were simplified to H atoms.

### Computational details for ORR

The VASP (*88, 89*) was employed to perform all DFT calculations within the generalized gradient approximation (GGA) with the Perdew-Burke-Ernzerhof (PBE) (*78*) formulation. The projected augmented wave (PAW) potentials (*90*) were used to describe the ionic cores. Valence electrons were taken into account using a plane wave basis set with a kinetic energy cutoff of 450 eV. Partial occupancies of the Kohn−Sham orbitals were allowed using the Gaussian smearing method and a width of 0.05 eV. The electronic energy was considered self-consistent when the energy change was smaller than $10^{-5}$ eV. A geometry optimization was considered convergent when the force change was smaller than 0.03 eV/Å. The Brillouin zone was sampled with 1×1×1 Gamma mesh (*91*).

### Gibbs free energies

Gibbs free energy can be obtained by adding corrections including entropic (*TS*) and zero-point energy (ZPE) to the calculated DFT energy, so that

$$\Delta G = \Delta E_{DFT} + \Delta ZPE - T\Delta S - eU \quad \text{(S1)}$$

where $\Delta E_{DFT}$ is the calculated DFT reaction energy, $\Delta ZPE$ is the change in ZPE calculated from the vibrational frequencies and $\Delta S$ is the change in the entropy referring to thermodynamics

databases. The electrode potential was adopted with respect to the reversible hydrogen electrode (RHE), which makes the standard electrochemical potential of electron involved in reaction ($G_e$) equal to $-eU$, and the standard electrochemical potential of the proton ($G_{H+}$) equal to that of the hydrogen atom in gaseous $H_2$ ($1/2G_{H2}$).

**Electrochemical measurements**

Electrochemical experiments were conducted on a CHI 760E electrochemical workstation (Shanghai Chenhua Instrument Co., Ltd.) with a three-electrode system and a rotating ring-disk electrode setup (RRDE, Pine Instruments Corporation) under ambient conditions. An RRDE with a glassy carbon disk (0.2475 $cm^2$ area) and a platinum ring electrode (0.1866 $cm^2$ area) was used as the working electrode. For electrochemical testing, the $Pd_{11}$ clusters were loaded onto commercial carbon black. The catalyst ink was prepared by dispersing 1.0 mg of catalyst into 200 μL of ethanol and 20 μL of Nafion solution (5.0 wt%), followed by ultrasonication for 1 hour to get homogeneous ink. Subsequently, 10 μL of catalyst ink was dropped and dried naturally on the polished glassy carbon electrode surface. An Ag/AgCl electrode and Pt wire were used as the reference and counter electrode, respectively. 0.1 M KOH (pH 13) was used as the electrolyte. All potentials of the electrochemical measurements were converted to RHE with the following equation:

$$E_{RHE} = E_{Ag/AgCl} + 0.059 \times pH + 0.197 \quad \text{(S2)}$$

Prior to the electrochemical tests, the electrolyte was saturated with oxygen by continuously passing $O_2$ through the electrolyte for 30 min. Subsequently, all the catalysts were electrochemically activated by cycling at 100 mV $s^{-1}$ until stable voltammograms were acquired. The LSV curves were recorded in $O_2$-saturated conditions with a scan rate of 10 mV $s^{-1}$ at 1600 rpm and the potential of the Pt ring electrode was set at 1.3 V (vs. RHE) to detect as-generated $H_2O_2$ on the disk electrode.

The hydrogen peroxide selectivity and electron transfer number (n) were calculated according to the following equations:

$$H_2O_2\ (\%) = 200 \times (I_r/N_c)/(|I_d| + I_r/N_c) \quad \text{(S3)}$$

$$n = 4 \times |I_d|/(|I_d| + I_r/N_c) \quad \text{(S4)}$$

where $I_r$ is the ring current, $I_d$ is the disk current, and $N_c$ is the collection efficiency of the RRDE (0.362 after calibration by $[Fe(CN)_6]^{3-/4-}$ redox system).

CV measurements were performed in 0.1 M KOH electrolyte over a potential range of 0.91–1.01 V (vs. RHE) at scan rates ranging from 10 to 50 mV $s^{-1}$, using the same three-electrode configuration as described above (working electrode: RRDE with glassy carbon disk and Pt ring; counter electrode: Pt wire; reference electrode: Ag/AgCl).

The EIS measurements were conducted under the same conditions by applying an AC voltage with an amplitude of 5 mV over a frequency range of $1\times10^{-2}$ to $5\times10^{5}$ Hz.

**Determination of $H_2O_2$ concentration**

The practical yield of $H_2O_2$ was measured using a two-compartment three-electrode flow cell reactor separated by an anion exchange membrane. Each compartment was filled with 50 mL of 0.1 M KOH. The $O_2$ flow rate was maintained at 20 sccm by using an electronic flowmeter throughout the electrolysis. The working electrode was prepared by drop-casting the **$Pd_{11}$-C** ink onto a gas-diffusion layer with a catalyst loading of 1.5 mg $cm^{-2}$. An Hg/HgO electrode and a Pt plate were used as the reference and counter electrodes, respectively. Bulk ORR electrolysis was conducted at various applied potentials for 30 min. $H_2O_2$ produced at each potential was quantified by the ceric sulfate titration method, based on the following reaction:

$$2Ce^{4+}+H_2O_2 \rightarrow 2Ce^{3+}+2H^{+}+O_2 \quad \text{(S5)}$$

where yellow-colored $Ce^{4+}$ can be reduced to the colorless $Ce^{3+}$ with the addition of $H_2O_2$, thus the yield of $H_2O_2$ can be calculated by the consumption of $Ce^{4+}$ according to the following equation:

$$C_{H_2O_2} = [V_{Ce^{4+}}\times C_{Ce^{4+}{}_{before}}-(V_{Ce^{4+}}+V_{electrolyte})\times C_{Ce^{4+}{}_{after}}]/(2\times V_{electrolyte}) \quad \text{(S6)}$$

where $V_{Ce^{4+}}$ is the volume of the added $Ce(SO_4)_2$ solution, $C_{Ce^{4+}{}_{before}}$ and $C_{Ce^{4+}{}_{after}}$ represent the concentration of $Ce^{4+}$ before and after the reaction, respectively, and $V_{electrolyte}$ is the volume of injected electrolyte after reaction. The standard concentration-absorbance curve was obtained by linearly fitting the absorbance values at 316 nm for a series of known $Ce^{4+}$ concentrations. The faradaic efficiency (FE) was calculated according to the following equation:

$$FE\,(\%) = (2\times C\times V\times F)/Q\times 100\% \quad \text{(S7)}$$

where $C$ is the concentration of generated $H_2O_2$ (mol $L^{-1}$); $V$ is the volume of electrolyte (0.05 L), $F$ is the Faraday constant (96485 C $mol^{-1}$), and $Q$ is the passed charge during the electrolysis (C).

**Operando ATR-SEIRAS measurements**

ATR-SEIRAS experiments were performed on a Bruker Vertex 70 equipped with a liquid nitrogen cooled MCT detector and a Veemax III ATR accessory (Kromatek) and a homemade three-electrode electrochemical cell. The 0.1 M KOH solution was used as the electrolyte. A Pt plate and

saturated Ag/AgCl were used as the counter electrode and reference electrode, respectively. The ATR-SEIRAS spectra were obtained by OPUS software. All spectra were collected at a 4 $cm^{-1}$ spectral resolution.

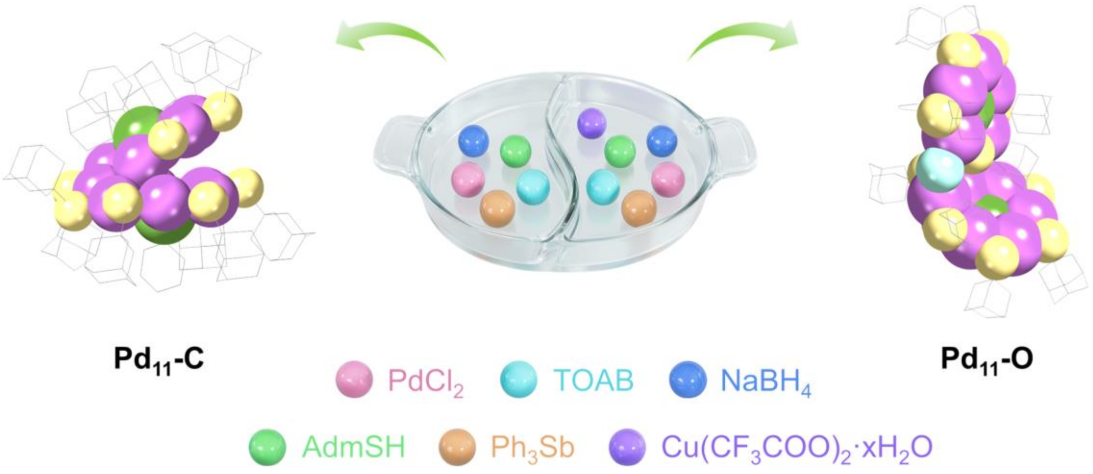


**Fig. S1. Synthetic illustrations of $Pd_{11}$-C and $Pd_{11}$-O clusters.** Color labels: purple, Pd; yellow, S; green, Sb; blue, Cl; gray, C. All hydrogen atoms are omitted for clarity.

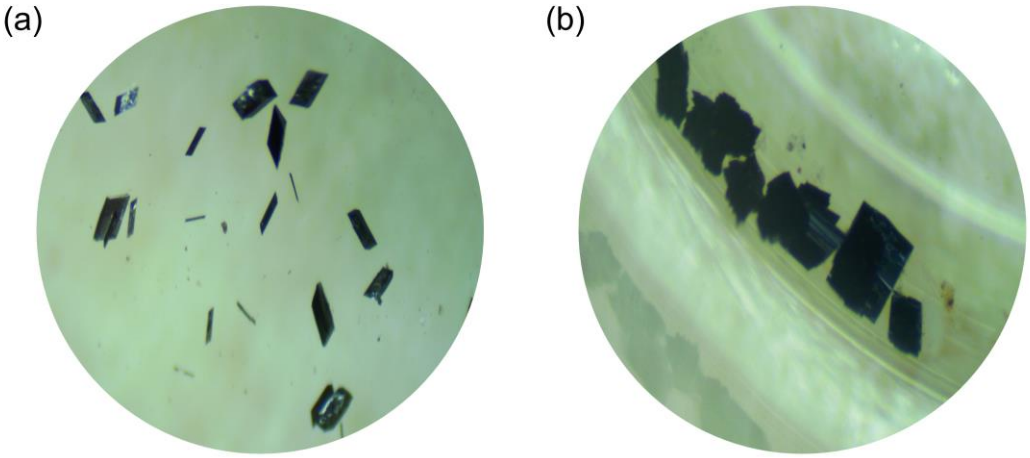

**Fig. S2. The photographs of $Pd_{11}$-C (a) and $Pd_{11}$-O (b) single crystals.**

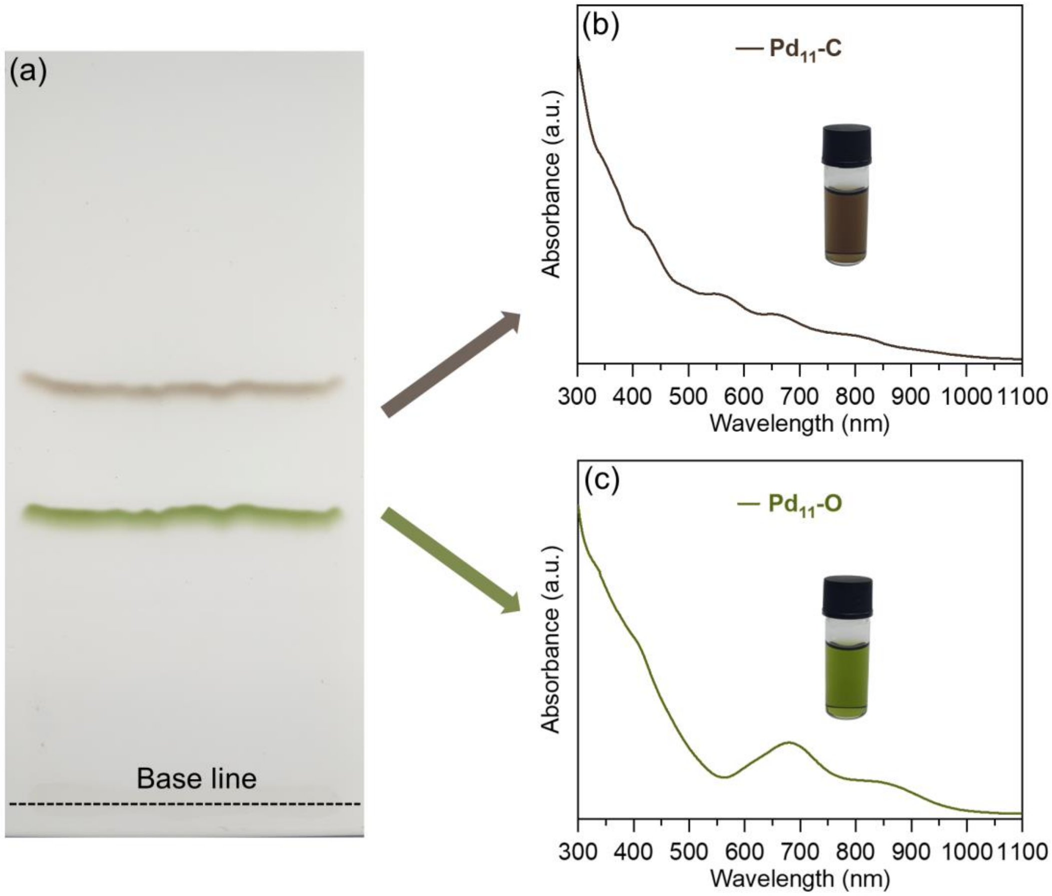


**Fig. S3. The positions on PTLC plate and optical absorption properties of $Pd_{11}$-C and $Pd_{11}$-O clusters.** (a) Photograph of the PTLC plate used for distinguishing **$Pd_{11}$-C** and **$Pd_{11}$-O** clusters; UV–Vis–NIR absorption spectra of **$Pd_{11}$-C** (b) and **$Pd_{11}$-O** (c) clusters (Insets: photos of the corresponding cluster solutions in dichloromethane).

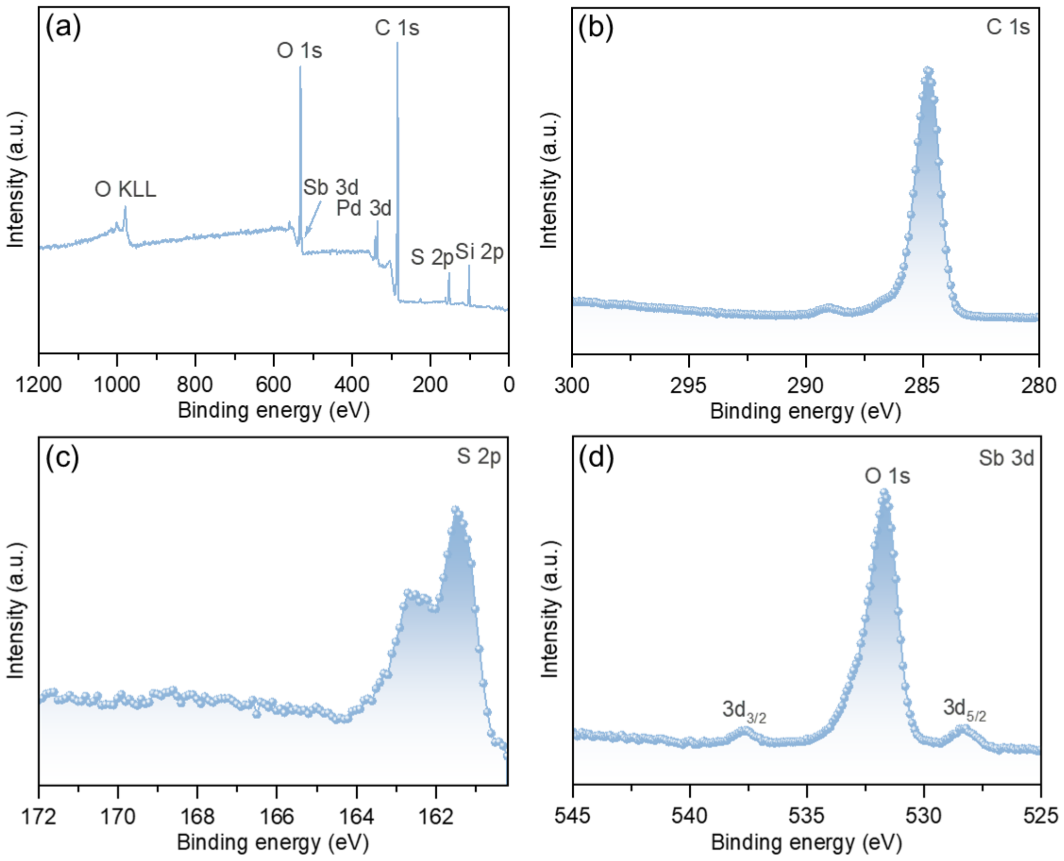


**Fig. S4. XPS spectra of $Pd_{11}$-C clusters.** (a) Survey spectrum of **$Pd_{11}$-C** clusters; (b–d) High-resolution XPS spectra for C 1s, S 2p, and Sb 3d. Note: O KLL denotes the O Auger peak.

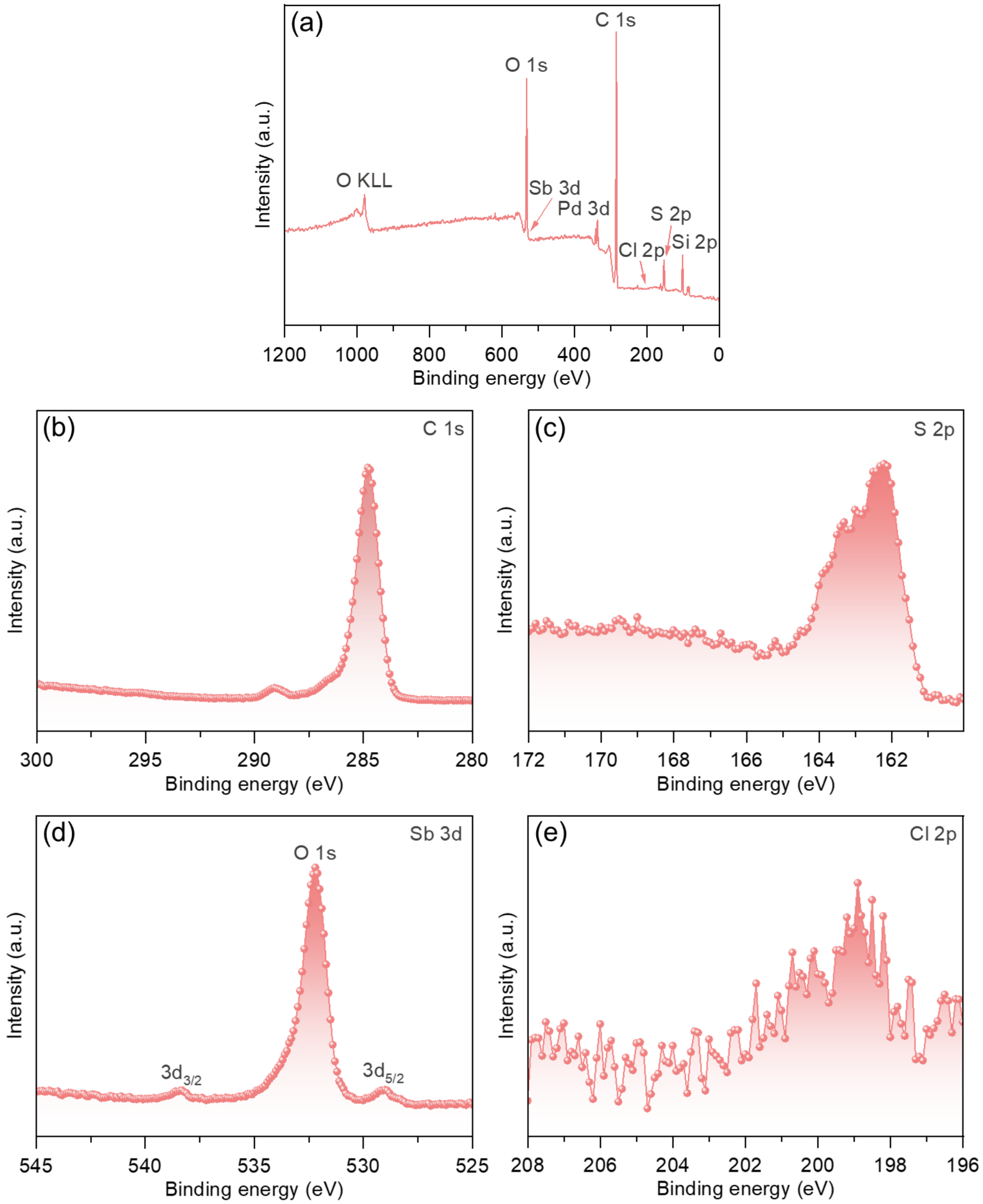


**Fig. S5. XPS spectra of $Pd_{11}$-O clusters.** (a) Survey spectrum of **$Pd_{11}$-O** clusters; (b–e) High-resolution XPS spectra for C 1s, S 2p, Sb 3d, and Cl 2p. Note: O KLL denotes the O Auger peak.

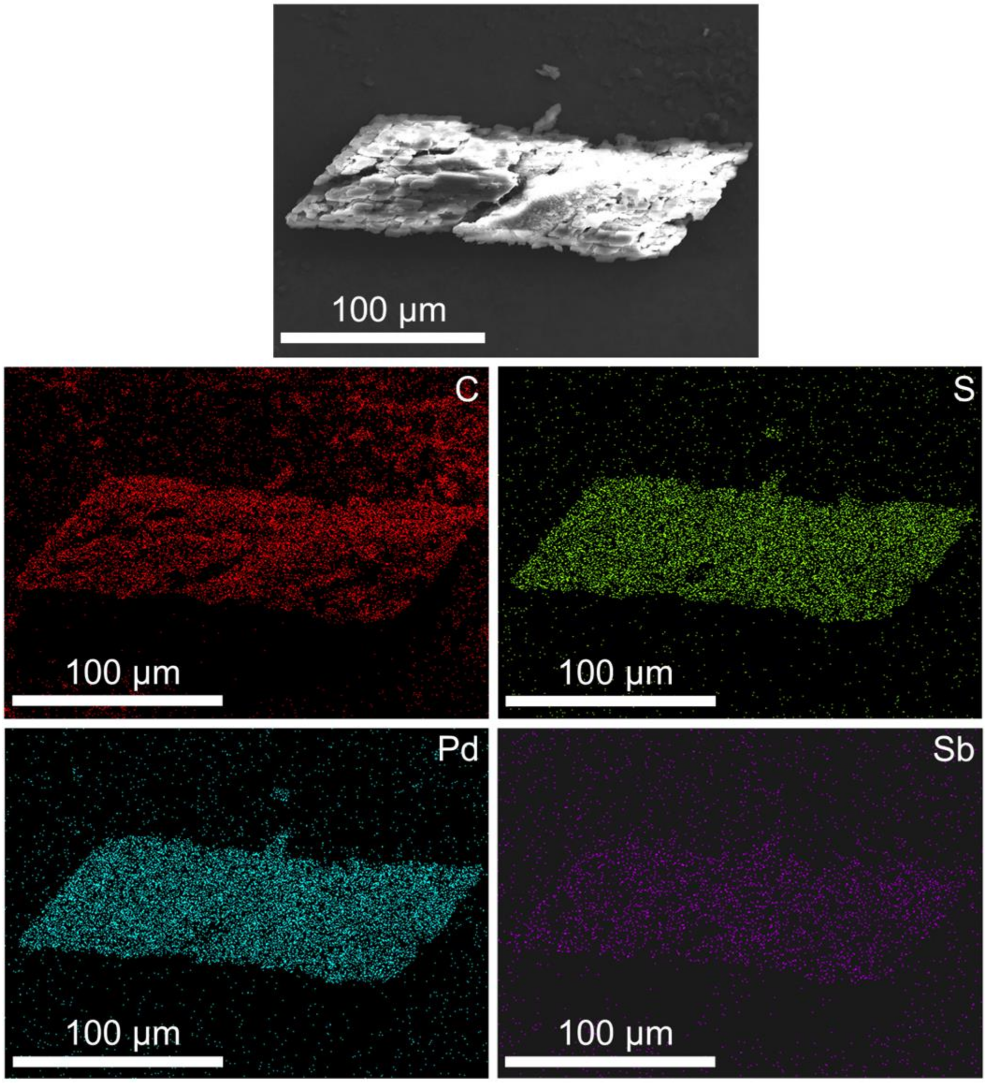


**Fig. S6. The EDS mapping of the crystalline $Pd_{11}$-C clusters.**

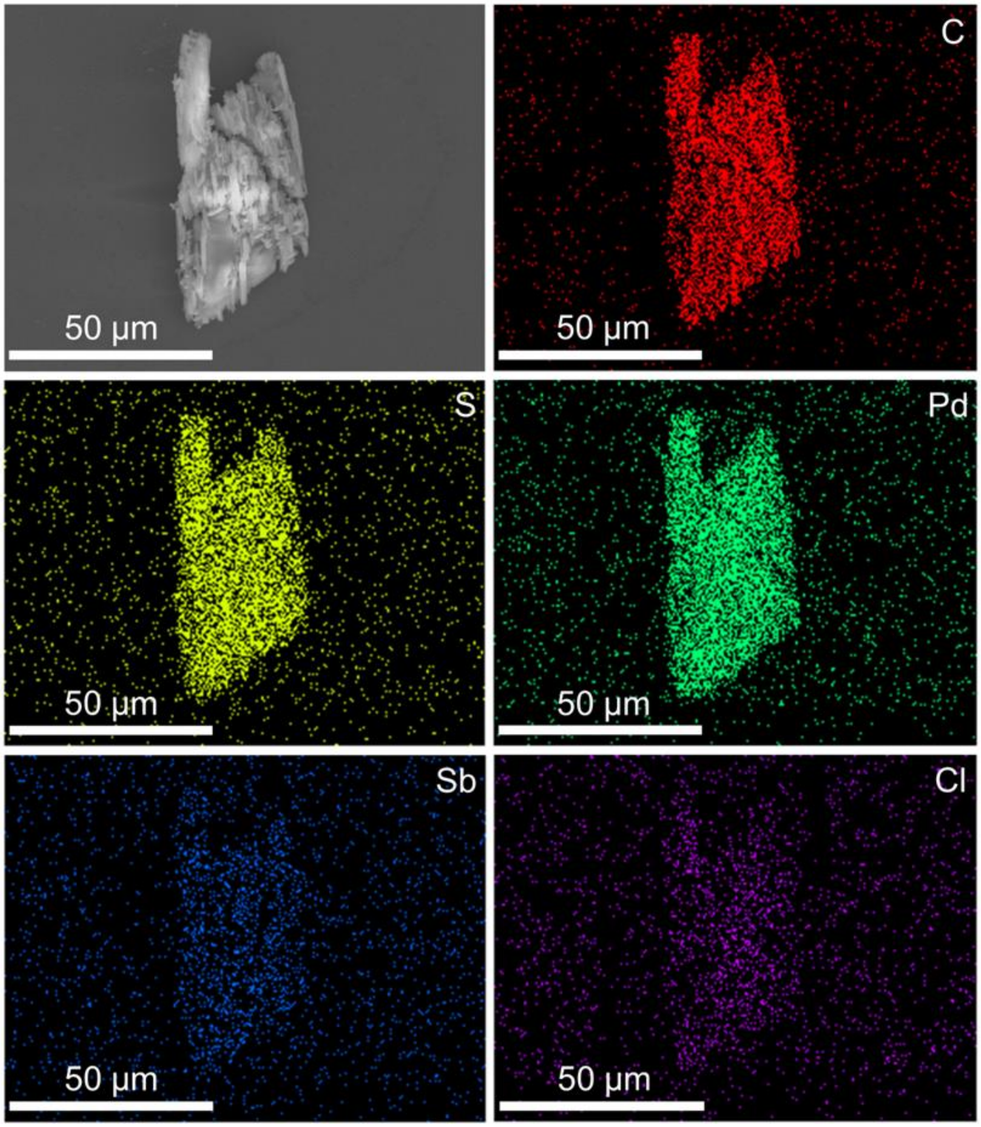


**Fig. S7. The EDS mapping of the crystalline $Pd_{11}$-O clusters.**

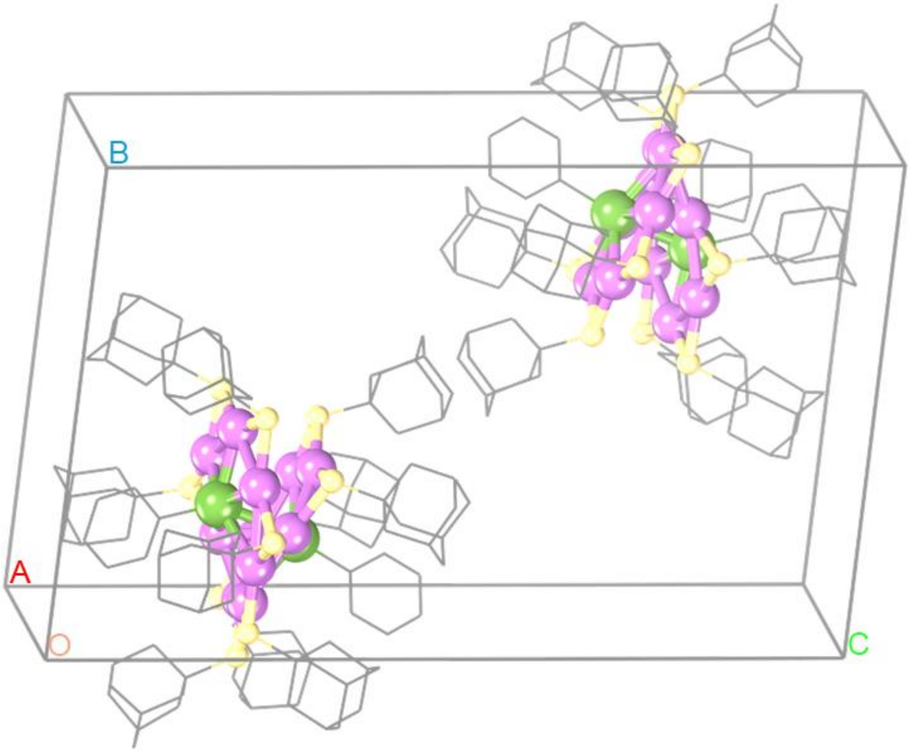


**Fig. S8. The unit cell of $Pd_{11}$-C.** Color labels: purple, Pd; yellow, S; green, Sb; gray, C.

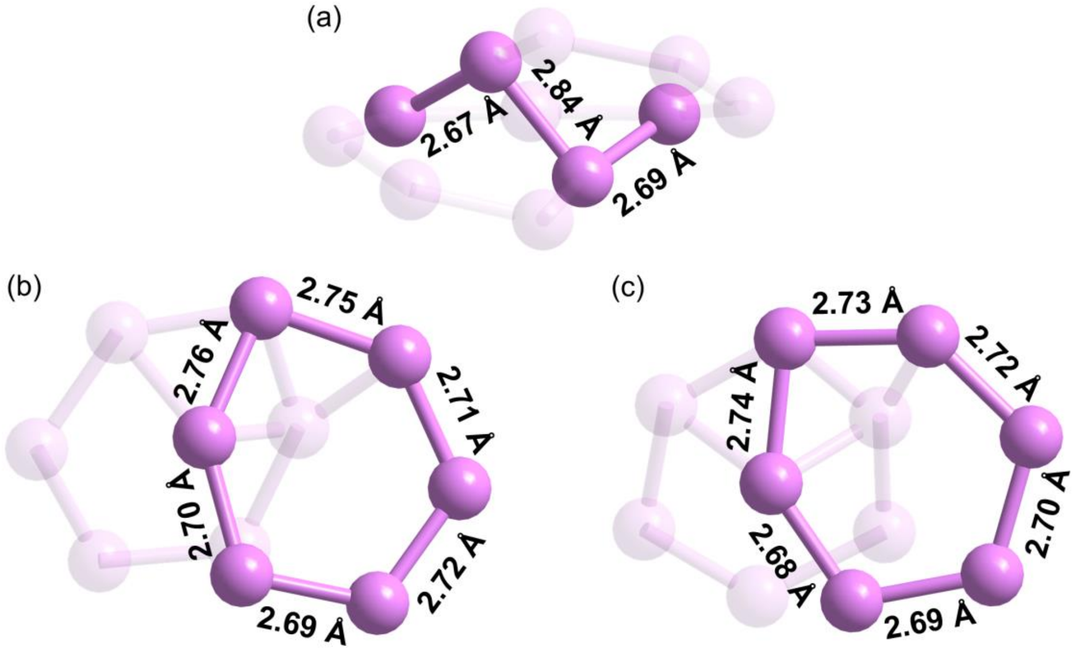


**Fig. S9. Side (a), top (b), and bottom (c) views of Pd–Pd bond lengths in the $Pd_{11}$-C cluster.** Color label: purple, Pd.

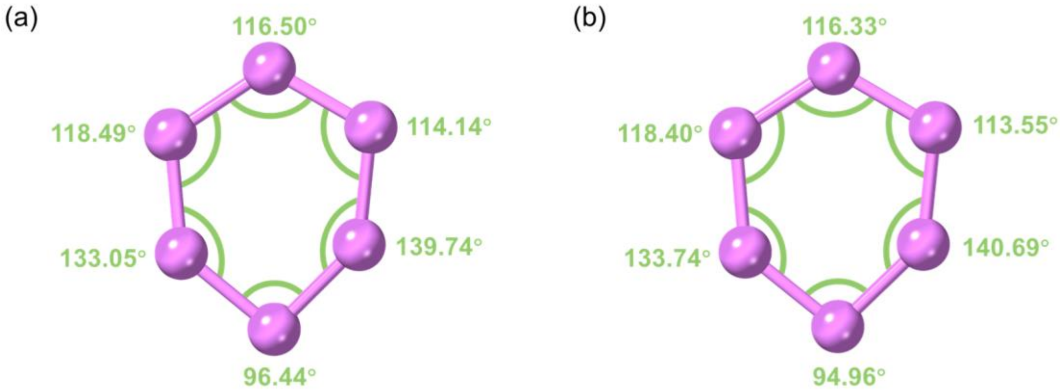


**Fig. S10. Comparison of Pd–Pd–Pd bond angles in the two $Pd_6$ rings of $Pd_{11}$-C cluster.** (a) One $Pd_6$ ring; (b) The other $Pd_6$ ring. Color label: purple, Pd.

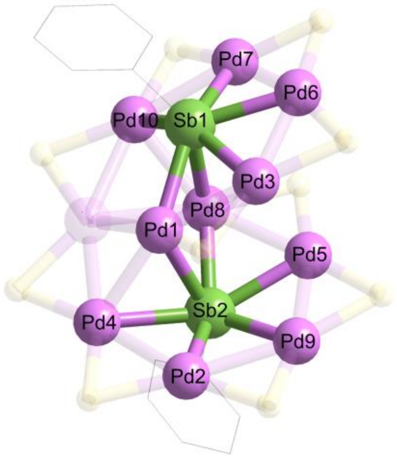


| Atom–Atom | Bond length/Å | Atom–Atom | Bond length/Å |
|---|---|---|---|
| Sb1–Pd1 | 2.64 | Sb2–Pd1 | 2.71 |
| Sb1–Pd3 | 2.65 | Sb2–Pd2 | 2.59 |
| Sb1–Pd6 | 2.59 | Sb2–Pd4 | 2.72 |
| Sb1–Pd7 | 2.59 | Sb2–Pd5 | 2.64 |
| Sb1–Pd8 | 2.70 | Sb2–Pd8 | 2.63 |
| Sb1–Pd10 | 2.72 | Sb2–Pd9 | 2.60 |

Note: The mean distance of Sb─Pd bonds is 2.65 Å.

**Fig. S11. Sb–Pd bond lengths in the $Pd_{11}$-C cluster.** Color labels: purple, Pd; yellow, S; green, Sb; gray, C. All hydrogen atoms and adamantanethiol ligands are omitted for clarity.

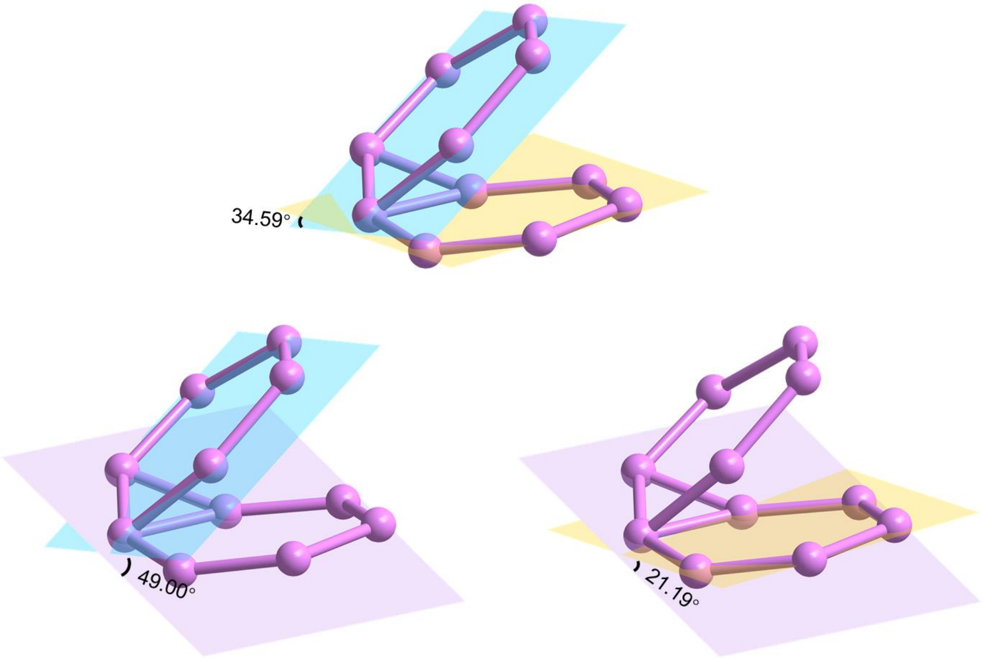


**Fig. S12. Dihedral angles between the $Pd_6$ rings and the $Pd_3$ triangle in the $Pd_{11}$ framework.** Color label: purple, Pd.

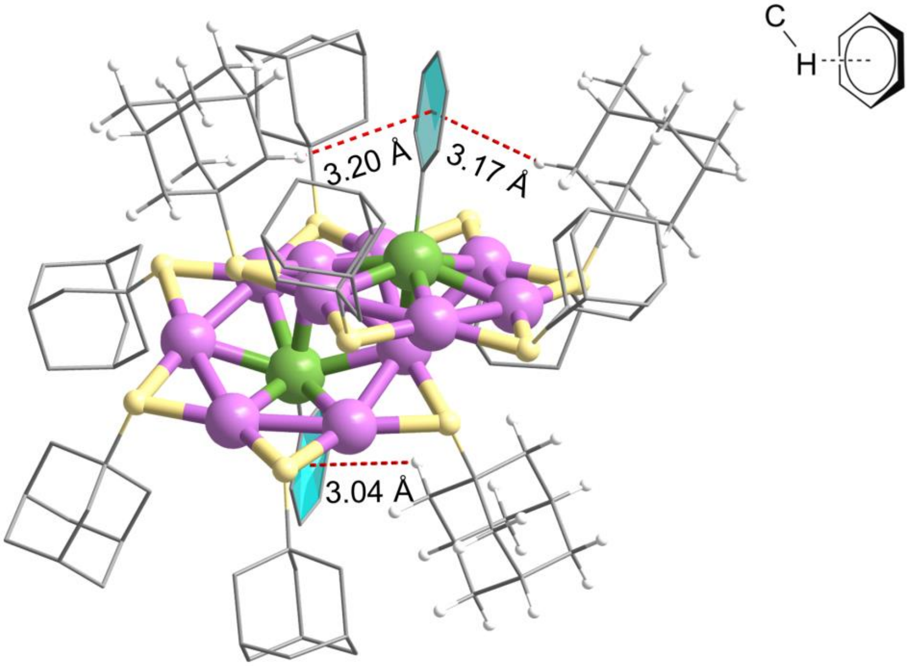

**Fig. S13. Intramolecular C–H···π interactions in the $Pd_{11}$-C cluster.** Color labels: purple, Pd; yellow, S; green, Sb; gray, C; white, H.

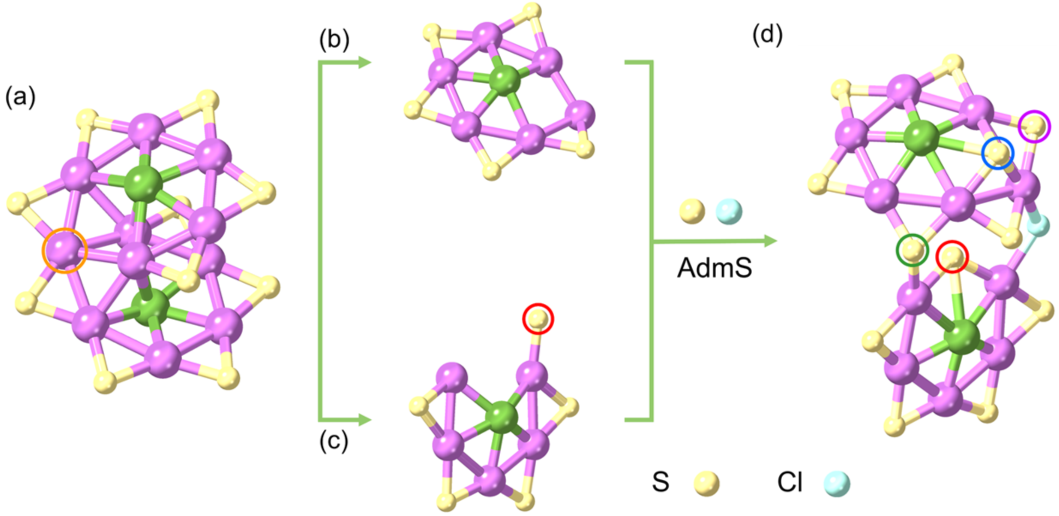


**Fig. S14. Analysis of the structural evolution from $Pd_{11}$-C to $Pd_{11}$-O cluster.** (a) $Pd_{11}Sb_2S_{10}$ framework; (b) $Pd_6SbS_5$ subunit; (c) $Pd_5SbS_5$ subunit; (d) $Pd_{11}SClSb_2S_{11}$ framework. Color labels: purple, Pd; yellow, S; green, Sb; blue, Cl.

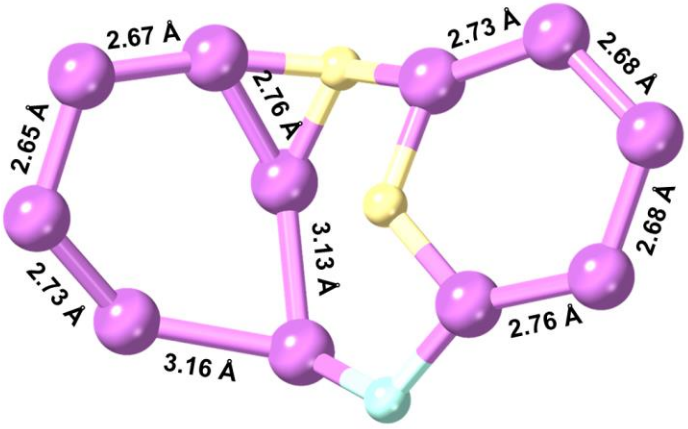


**Fig. S15. Pd–Pd bond lengths in the $Pd_{11}$-O cluster.** Color labels: purple, Pd; yellow, S; blue, Cl.

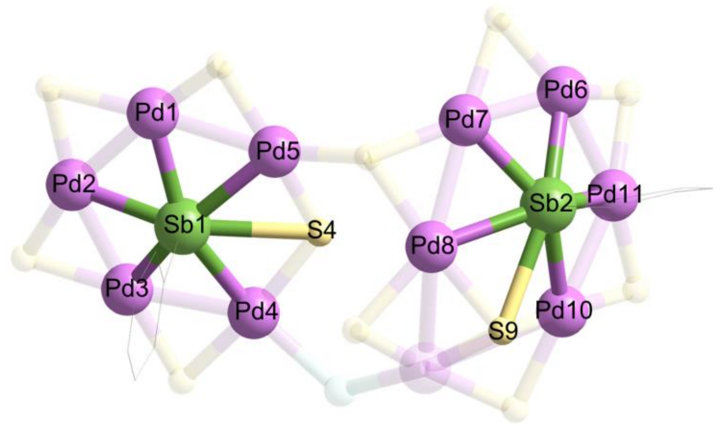


| Atom–Atom | Bond length/Å | Atom–Atom | Bond length/Å |
|---|---|---|---|
| Sb1–Pd1 | 2.52 | Sb2–Pd6 | 2.61 |
| Sb1–Pd2 | 2.61 | Sb2–Pd7 | 2.56 |
| Sb1–Pd3 | 2.54 | Sb2–Pd8 | 2.74 |
| Sb1–Pd4 | 2.65 | Sb2–Pd10 | 2.71 |
| Sb1–Pd5 | 2.73 | Sb2–Pd11 | 2.55 |
| Sb1–S4 | 2.89 | Sb2–S9 | 2.83 |

Note: The mean distance of Sb–Pd bonds is 2.62 Å.

**Fig. S16. Sb–Pd and Sb–S bond lengths in the $Pd_{11}$-O cluster.** Color labels: purple, Pd; yellow, S; green, Sb; blue, Cl; gray, C. All hydrogen atoms and adamantanethiol ligands are omitted for clarity.

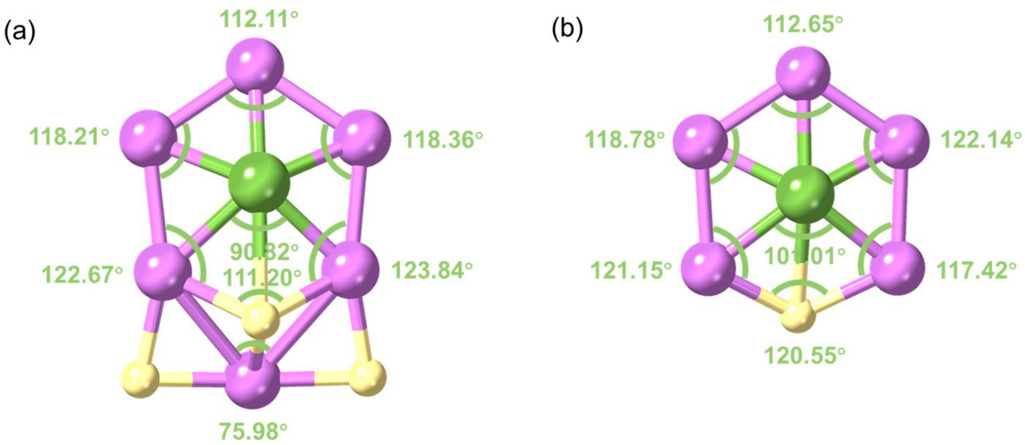


**Fig. S17. Comparison of bond angles in the two subunits of $Pd_{11}$-O cluster.** (a) $Pd_6SSbS_2$ subunit; (b) $Pd_5SbS$ subunit. Color labels: purple, Pd; yellow, S; green, Sb.

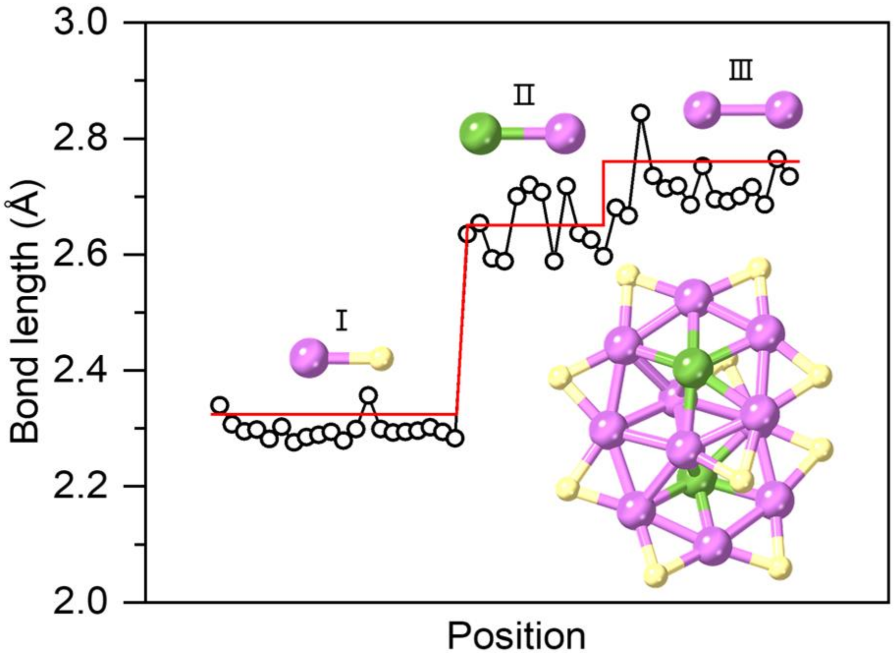


**Fig. S18. Bond lengths in the $Pd_{11}$-C cluster.** (I) Pd–S, (II) Sb–Pd, (III) Pd–Pd. Color labels: purple, Pd; yellow, S; green, Sb.

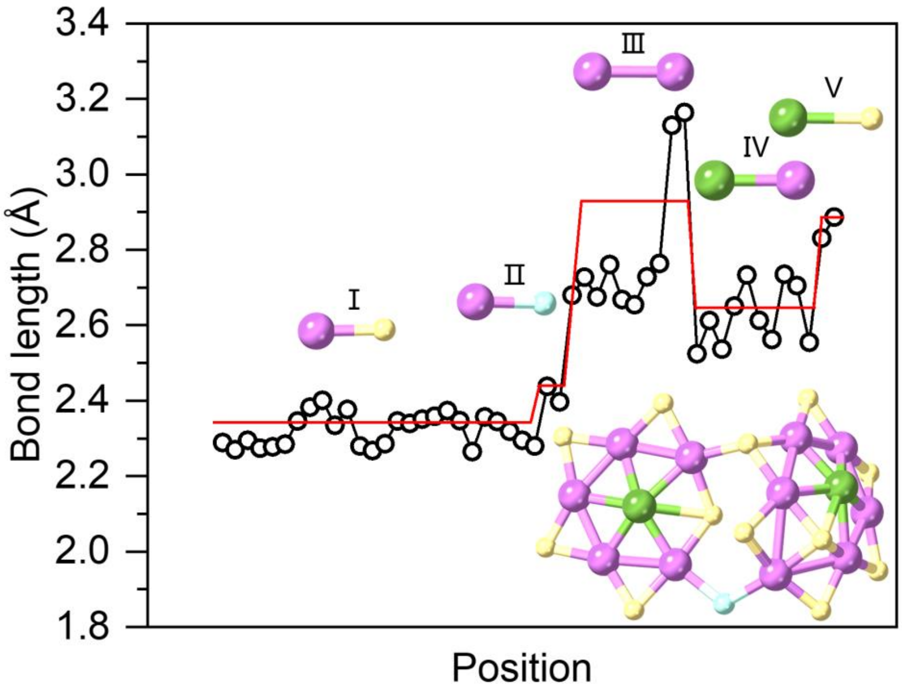


**Fig. S19. Bond lengths in the $Pd_{11}$-O cluster.** (I) Pd–S, (II) Pd–Cl, (III) Pd–Pd, (IV) Sb–Pd, (V) Sb–S. Color labels: purple, Pd; yellow, S; green, Sb; blue, Cl.

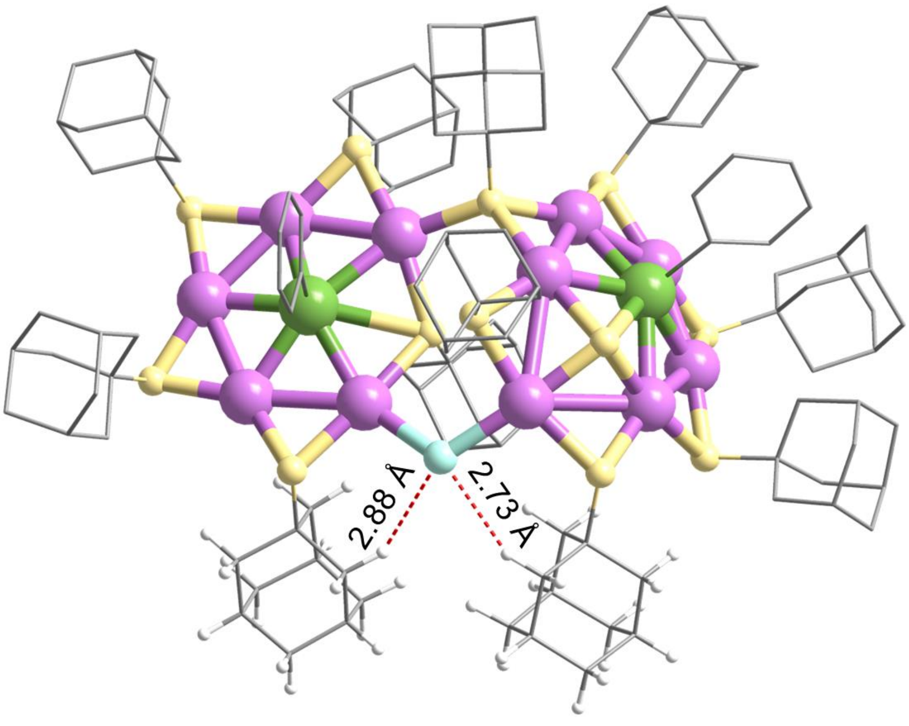


**Fig. S20. Intramolecular C–H···Cl interactions in the $Pd_{11}$-O cluster.** Color labels: purple, Pd; yellow, S; green, Sb; blue, Cl; gray, C; white, H.

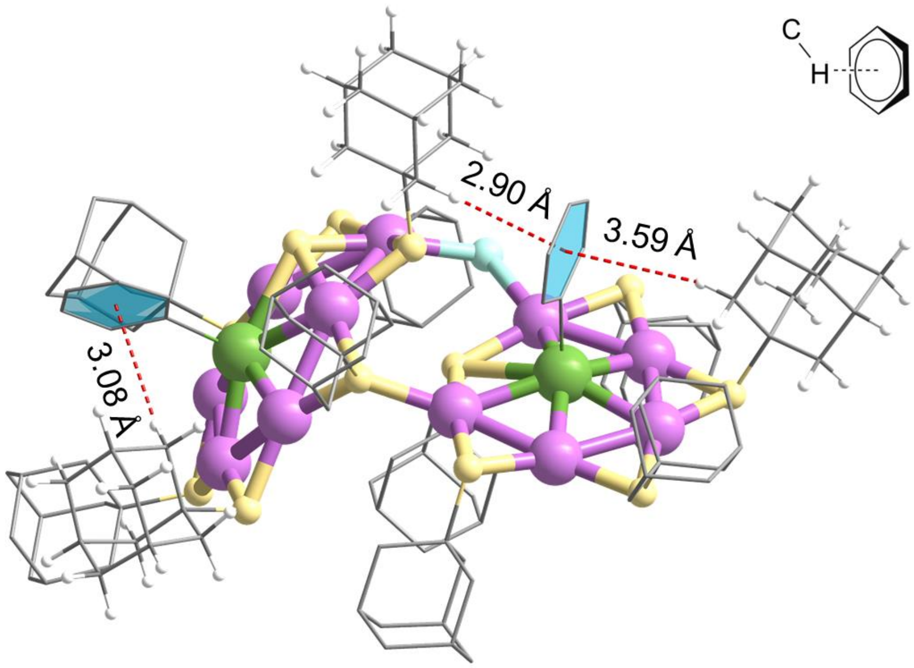


**Fig. S21. Intramolecular C–H···π interactions in the $Pd_{11}$-O cluster.** Color labels: purple, Pd; yellow, S; green, Sb; blue, Cl; gray, C; white, H.

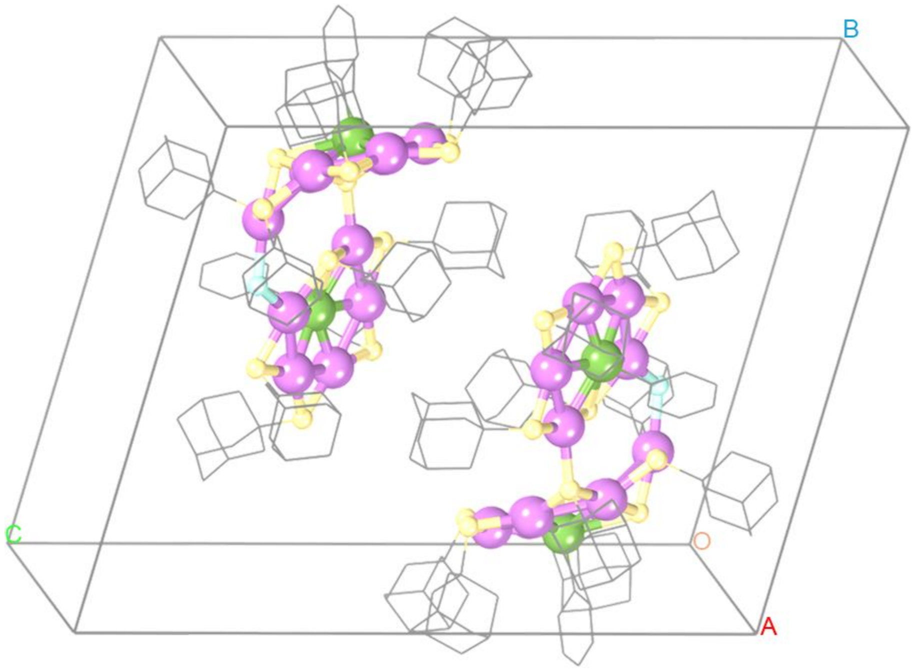


**Fig. S22. The unit cell of $Pd_{11}$-O.** Color labels: purple, Pd; yellow, S; green, Sb; blue, Cl; gray, C.

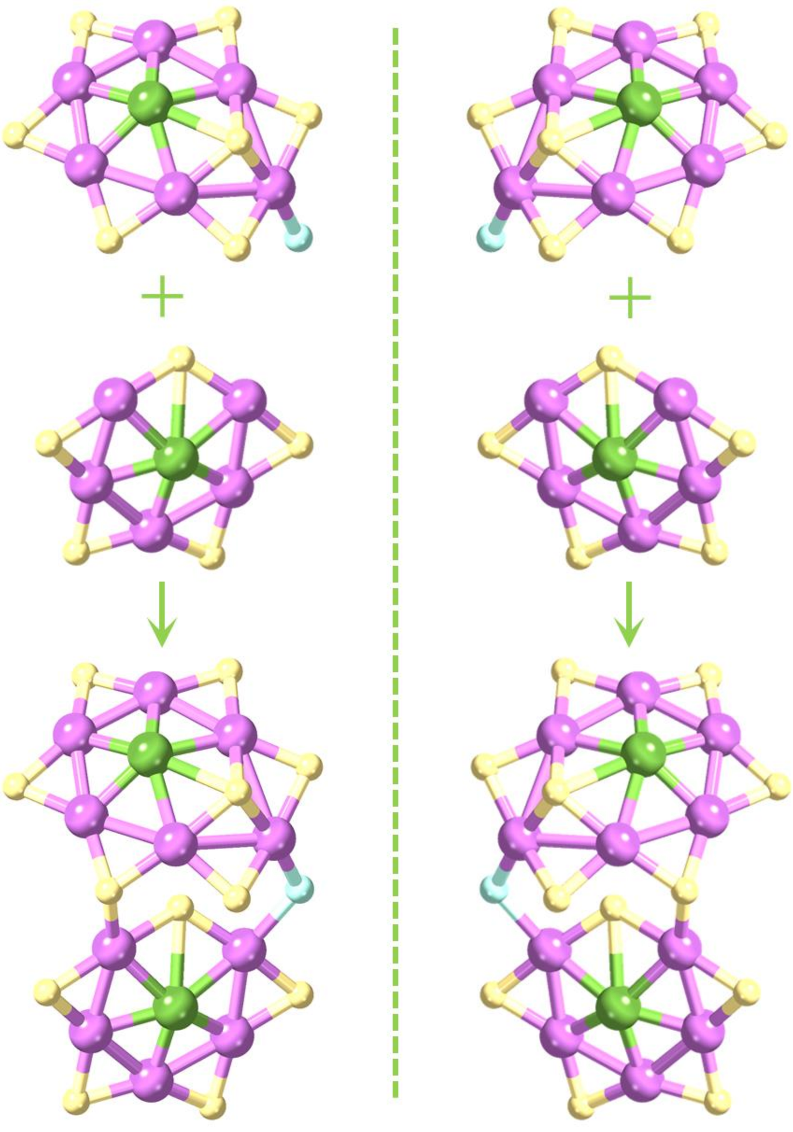


**Fig. S23. Structural anatomy of the two enantiomers in the unit cell of $Pd_{11}$-O clusters.** Color labels: purple, Pd; yellow, S; green, Sb; blue, Cl.

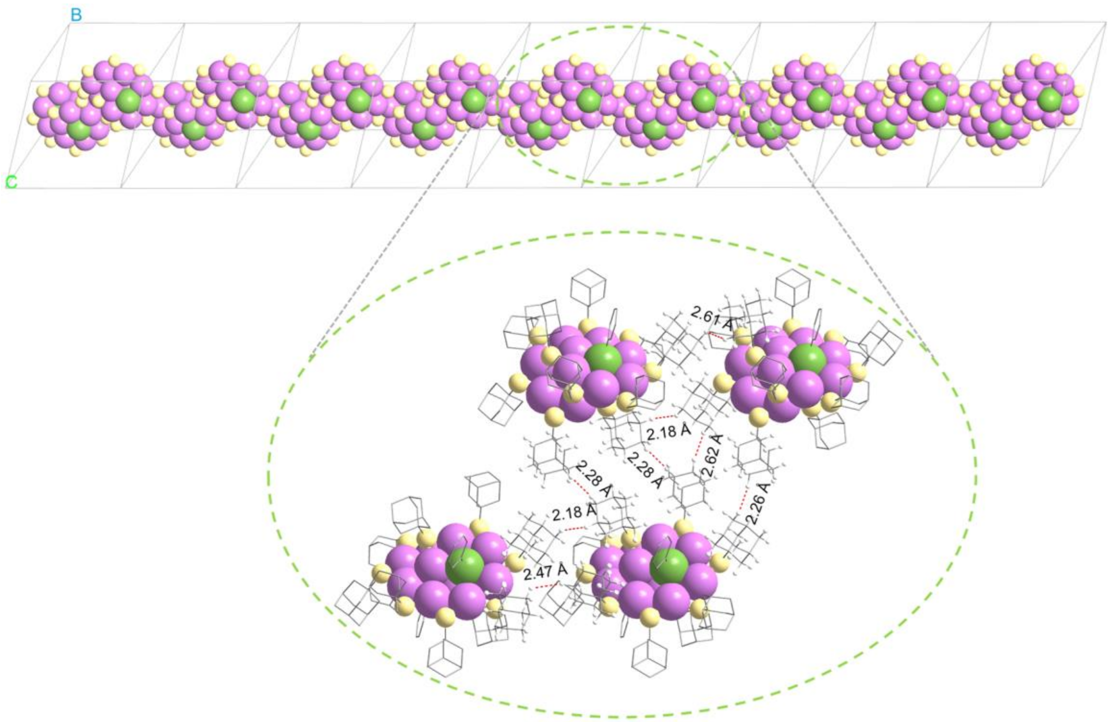


**Fig. S24. Intermolecular H···H interactions between neighboring $Pd_{11}$-C clusters.** Color labels: purple, Pd; yellow, S; green, Sb; gray, C; white, H.

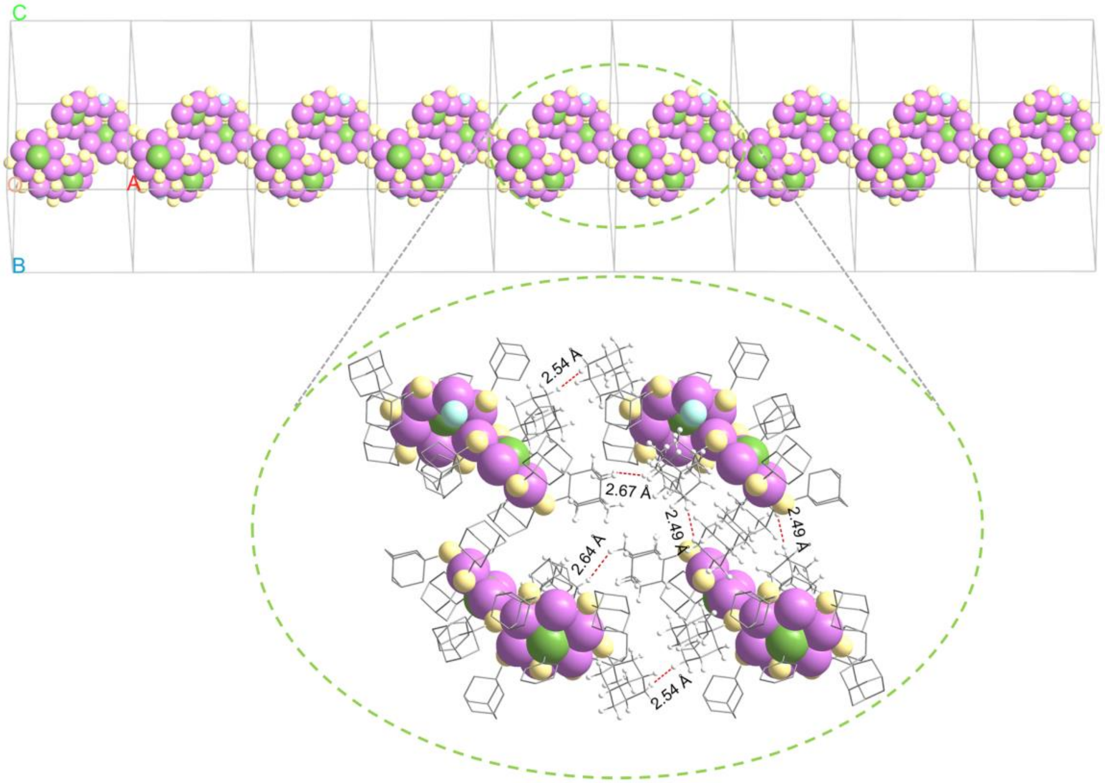


**Fig. S25. Intermolecular H⋯H interactions between neighboring $Pd_{11}$-O clusters.** Color labels: purple, Pd; yellow, S; green, Sb; blue, Cl; gray, C; white, H.

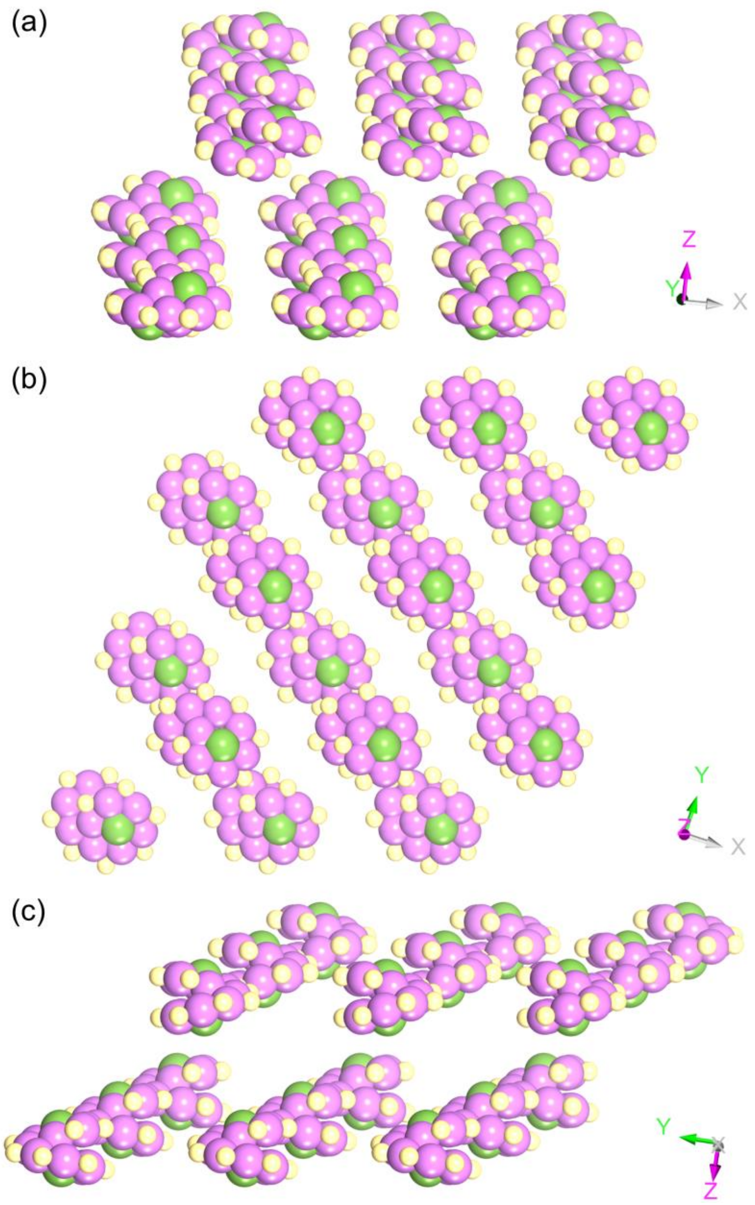


**Fig. S26. Space-filling view of the $Pd_{11}$-C clusters in 3 × 3 × 3 unit cells viewed along Y (a), Z (b), and X (c) axes.** All carbon and hydrogen atoms are omitted for clarity. Color labels: purple, Pd; yellow, S; green, Sb.

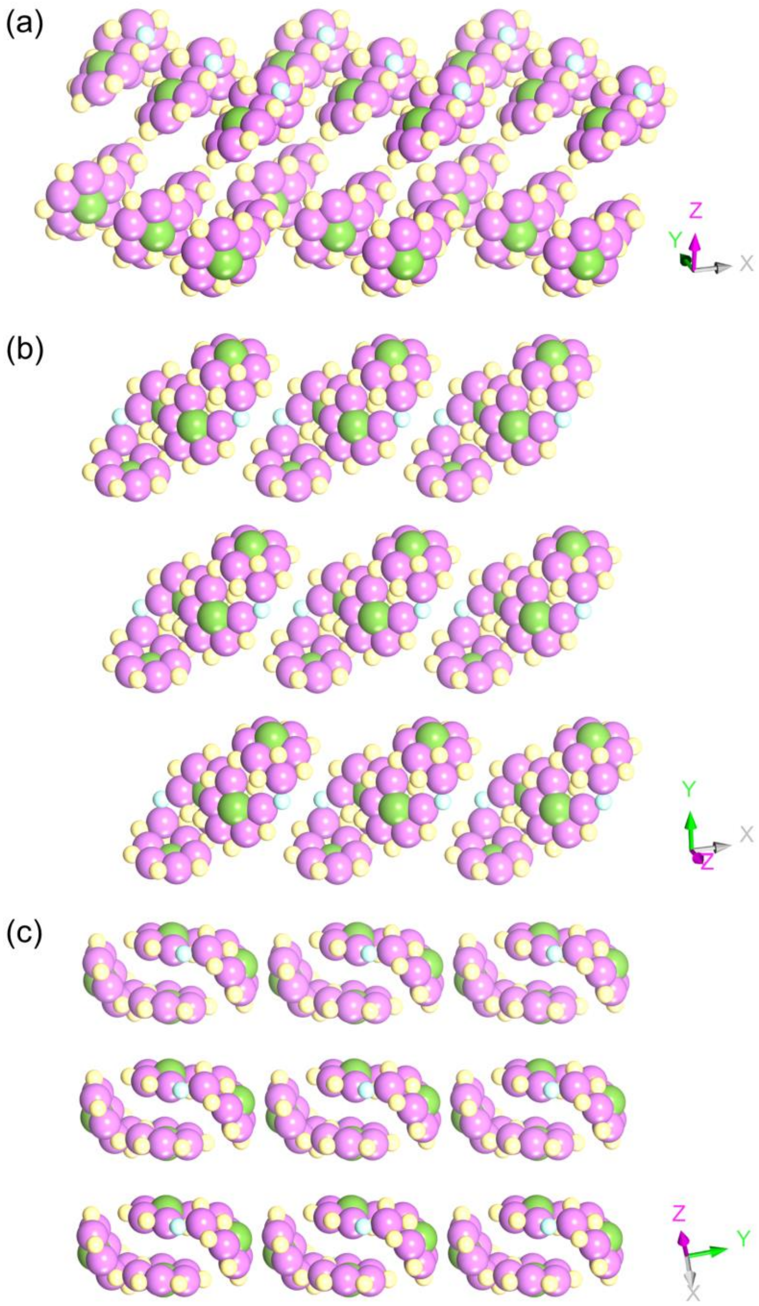


**Fig. S27. Space-filling view of the $Pd_{11}$-O clusters in 3 × 3 × 3 unit cells viewed along Y (a), Z (b), and X (c) axes.** All carbon and hydrogen atoms are omitted for clarity. Color labels: purple, Pd; yellow, S; green, Sb; blue, Cl.

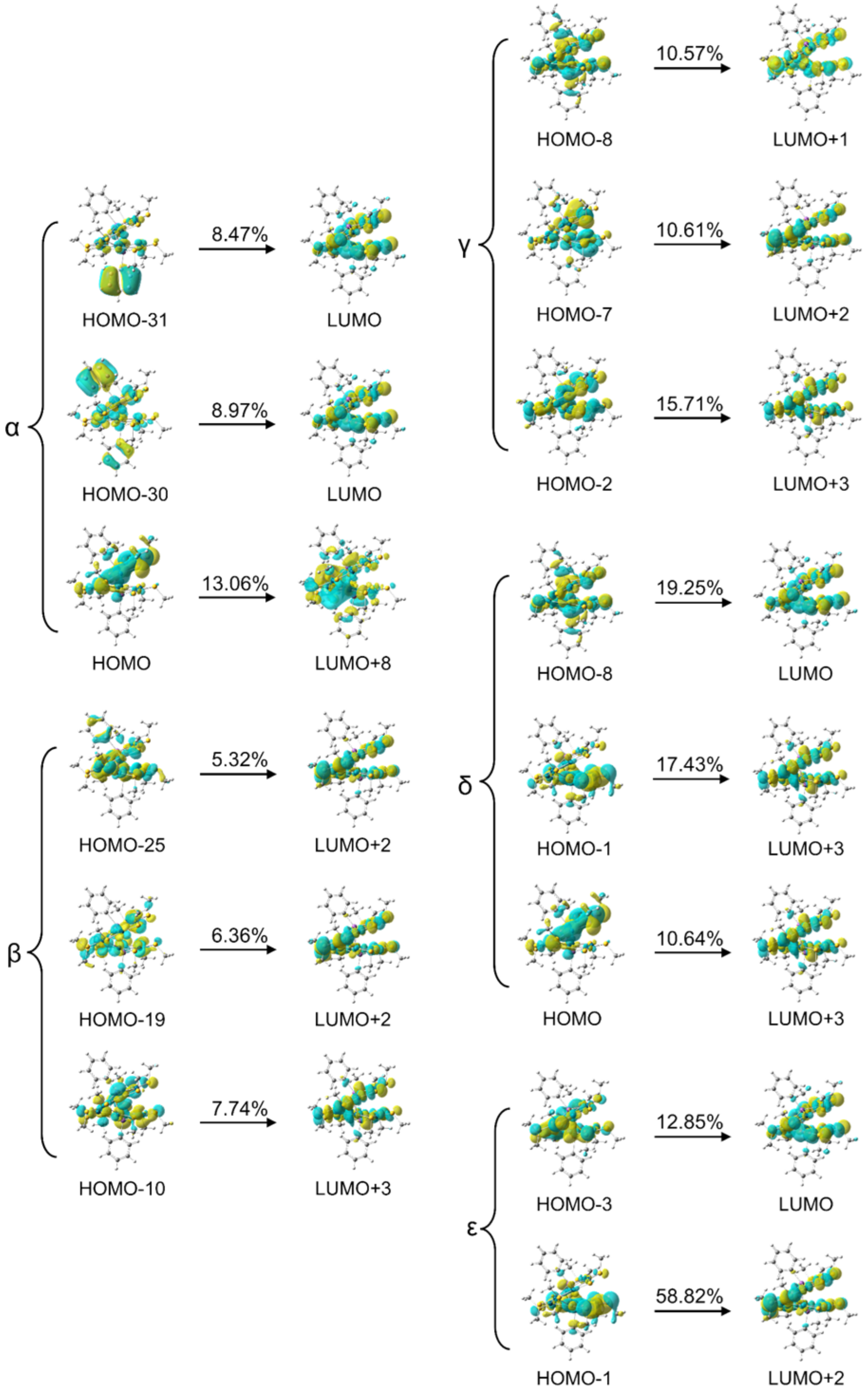


**Fig. S28. The computed electronic transitions are associated with the major absorption bands of $Pd_{11}$-C clusters.**

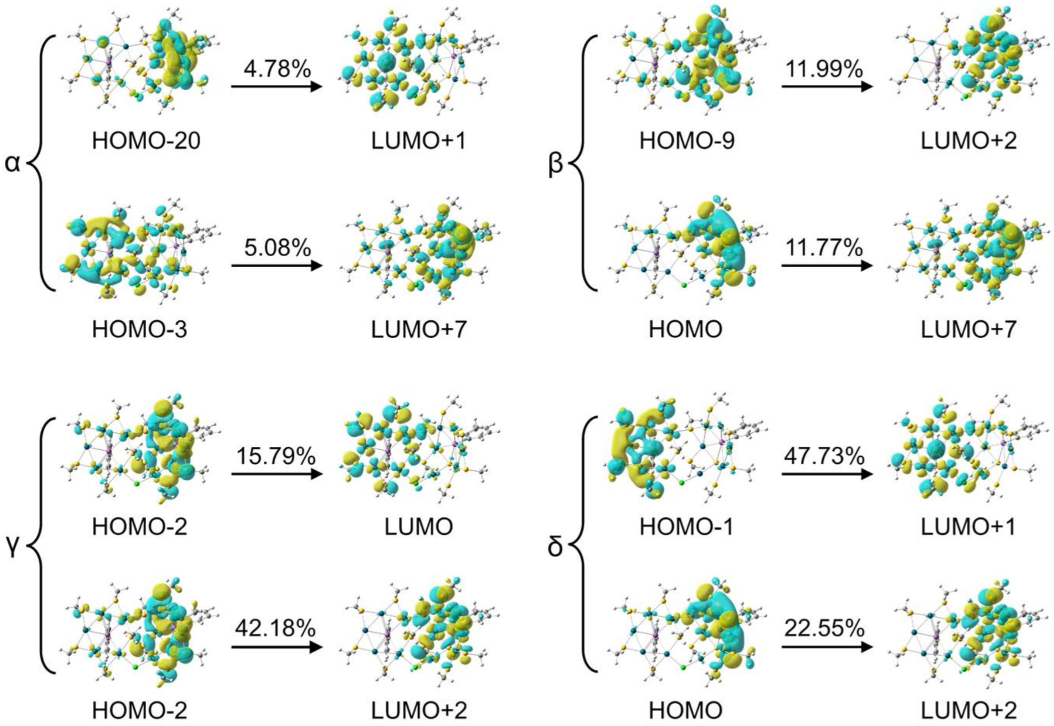


**Fig. S29. The computed electronic transitions are associated with the major absorption bands of $Pd_{11}$-O clusters.**

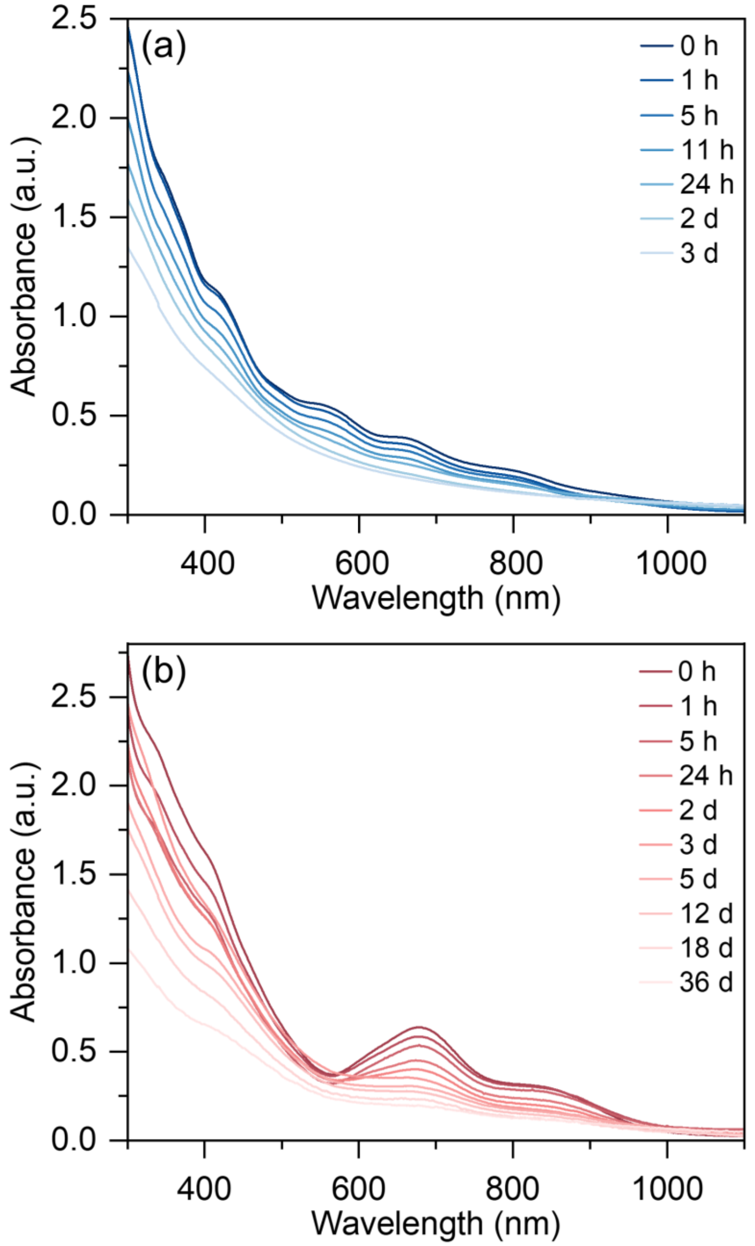


**Fig. S30.** **Time-dependent UV–Vis–NIR absorption spectra of $Pd_{11}$-C (a) and $Pd_{11}$-O (b) clusters in toluene at 80 °C.**

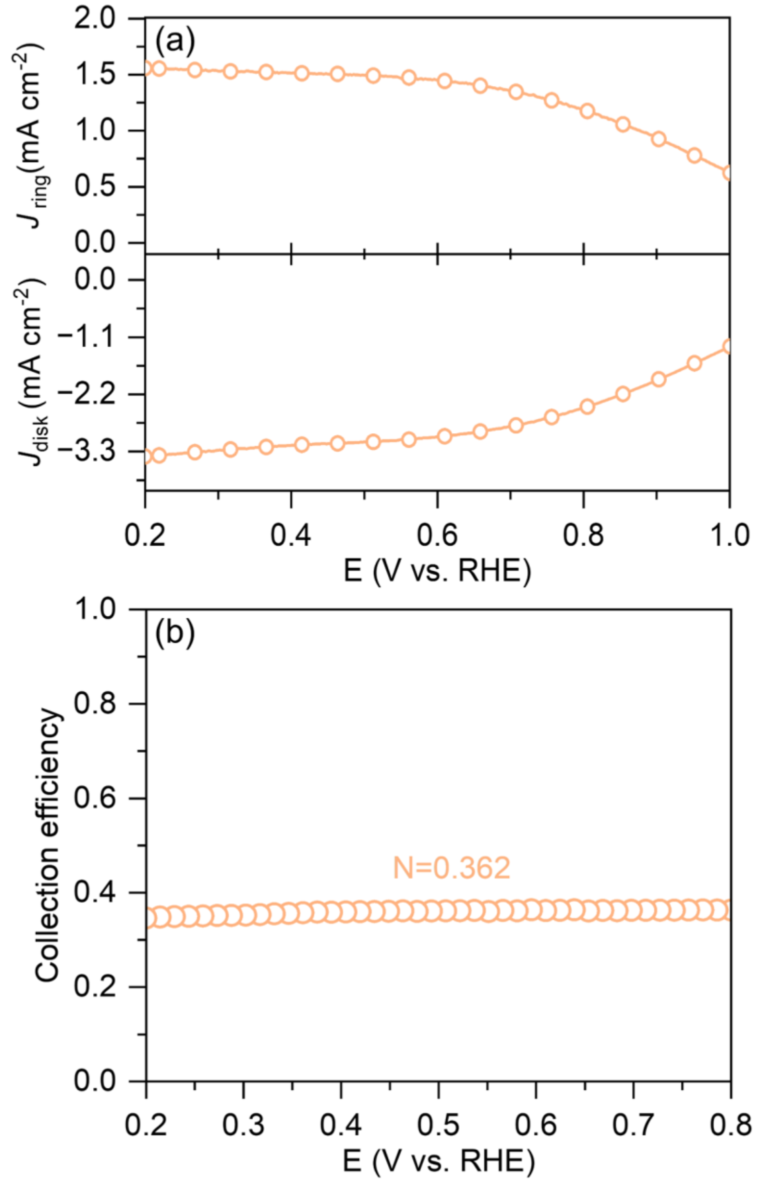


**Fig. S31. Calibration of the RRDE collection efficiency.** (a) LSV curves of the bare RRDE measured in $N_2$-saturated 0.1 M KOH containing 5.0 mM $K_3[Fe(CN)_6]$ collected at 1600 rpm; (b) Calibrated collection efficiency (N) obtained from the LSV data by dividing the ring current by the disk current.

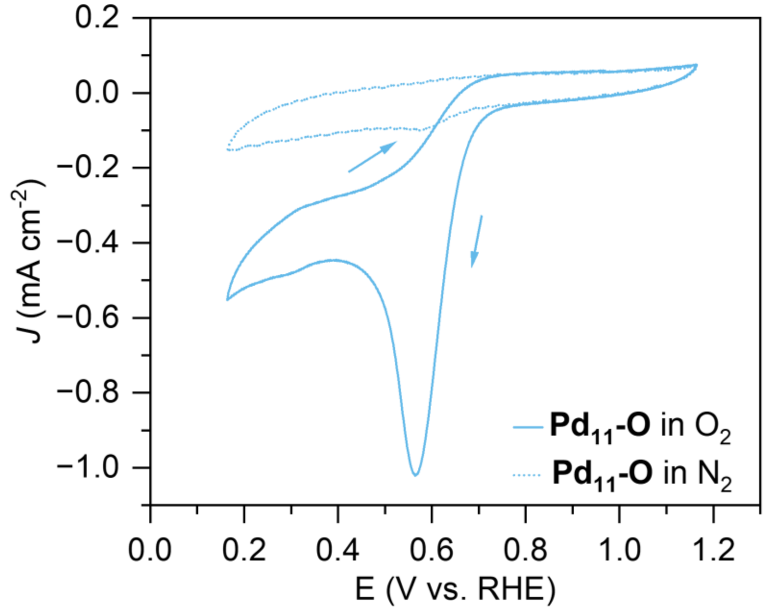


**Fig. S32. Cyclic voltammograms of $Pd_{11}$-O clusters in $O_2$-saturated and $N_2$-saturated 0.1 M KOH.**

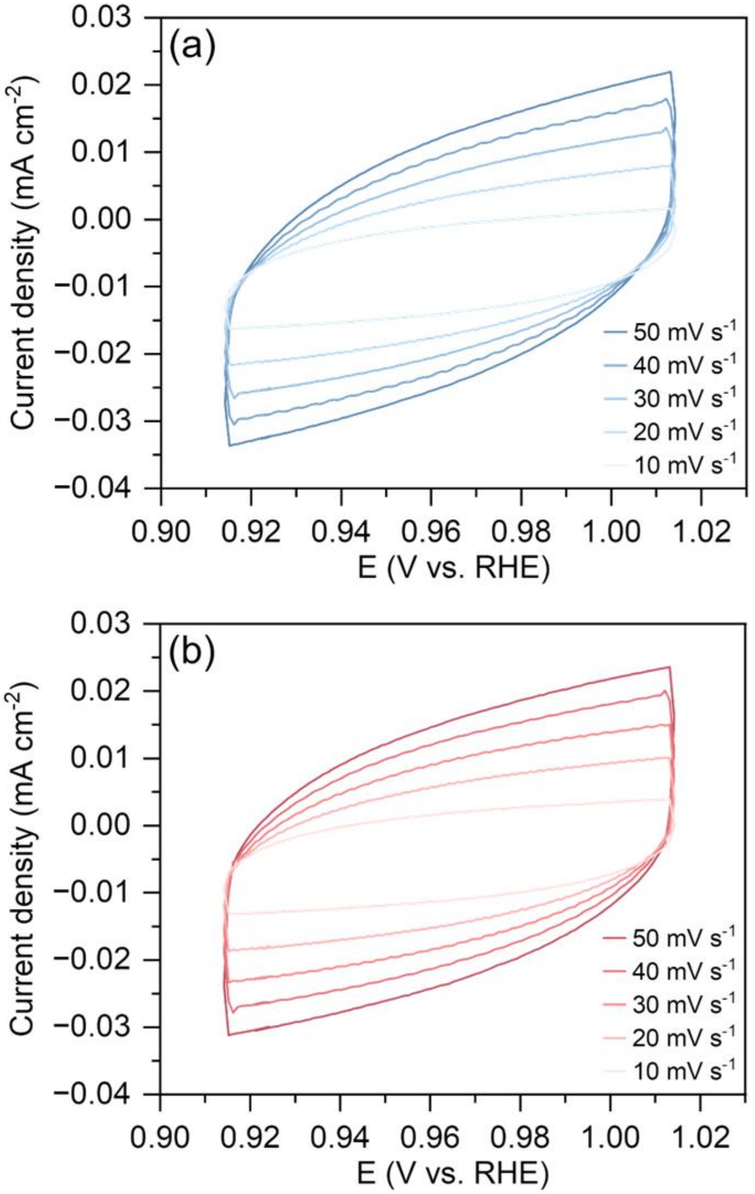


**Fig. S33. Cyclic voltammograms of $Pd_{11}$-C (a) and $Pd_{11}$-O (b) clusters at different scan rates.**

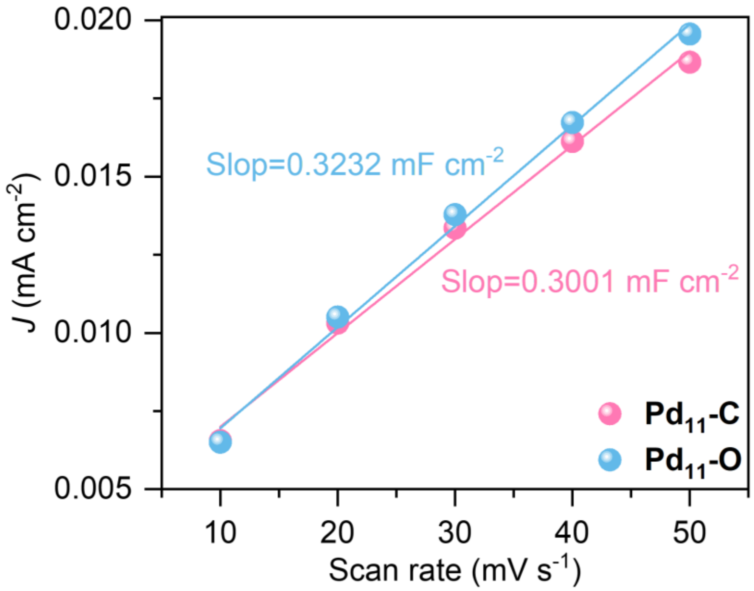


**Fig. S34. Double-layer capacitances ($C_{dl}$) of $Pd_{11}$ clusters derived from CV results at different scan rates.**

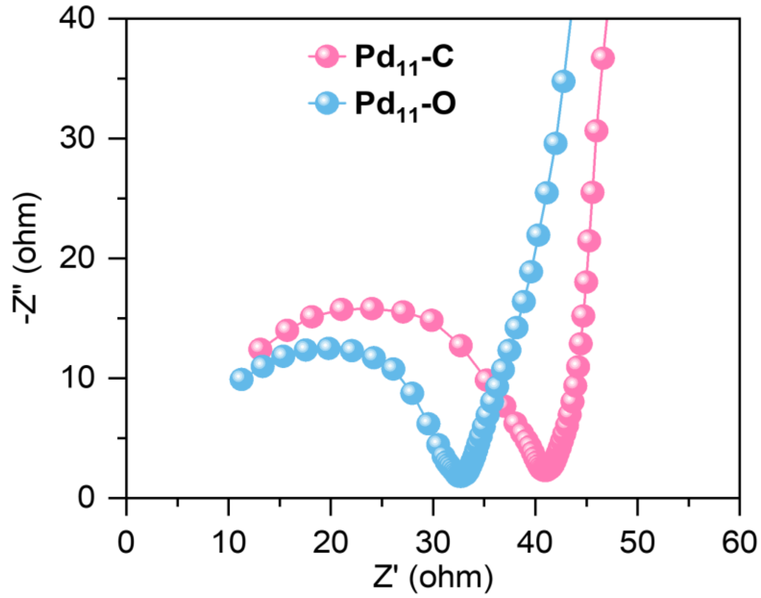


**Fig. S35. EIS curves of $Pd_{11}$ clusters.**

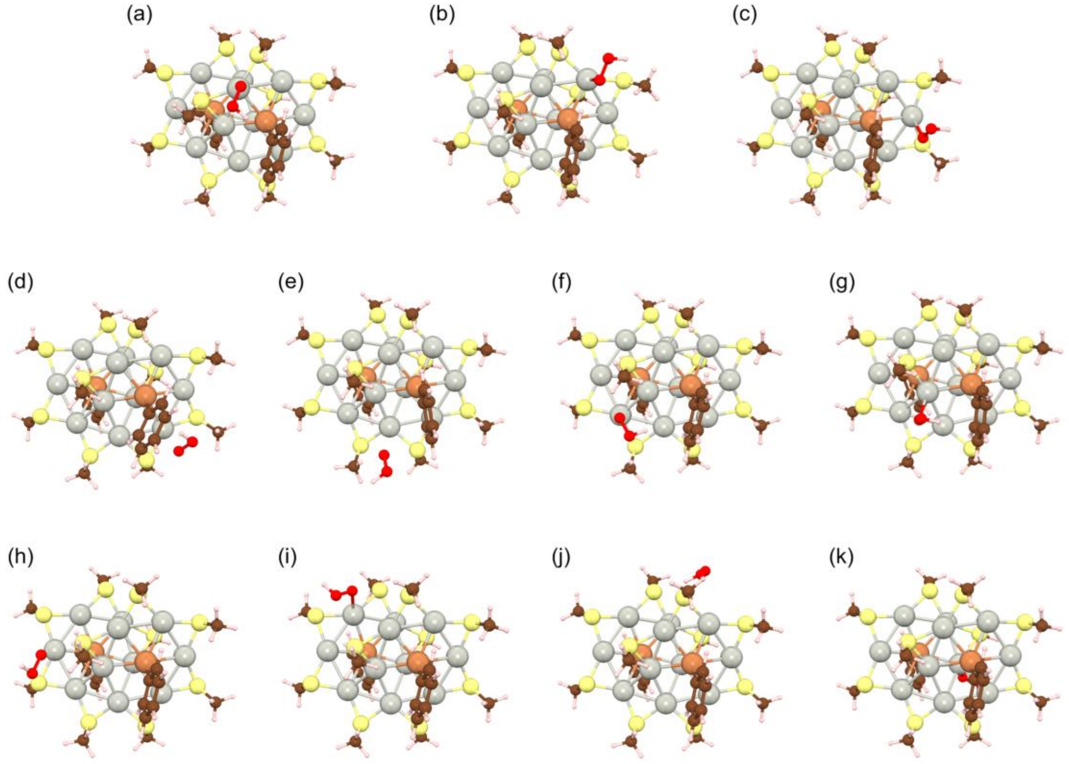


**Fig. S36. Rate-determining steps on possible structural sites of $Pd_{11}$-C clusters.** Pd1 site (a), Pd2 site (b), Pd3 site (c), Pd4 site (d), Pd5 site (e), Pd6 site (f), Pd7 site (g), Pd8 site (h), Pd9 site (i), Pd10 site (j), and Pd11 site (k). Color labels: gray, Pd; yellow, S; orange, Sb; red, O; brown, C; pink, H.

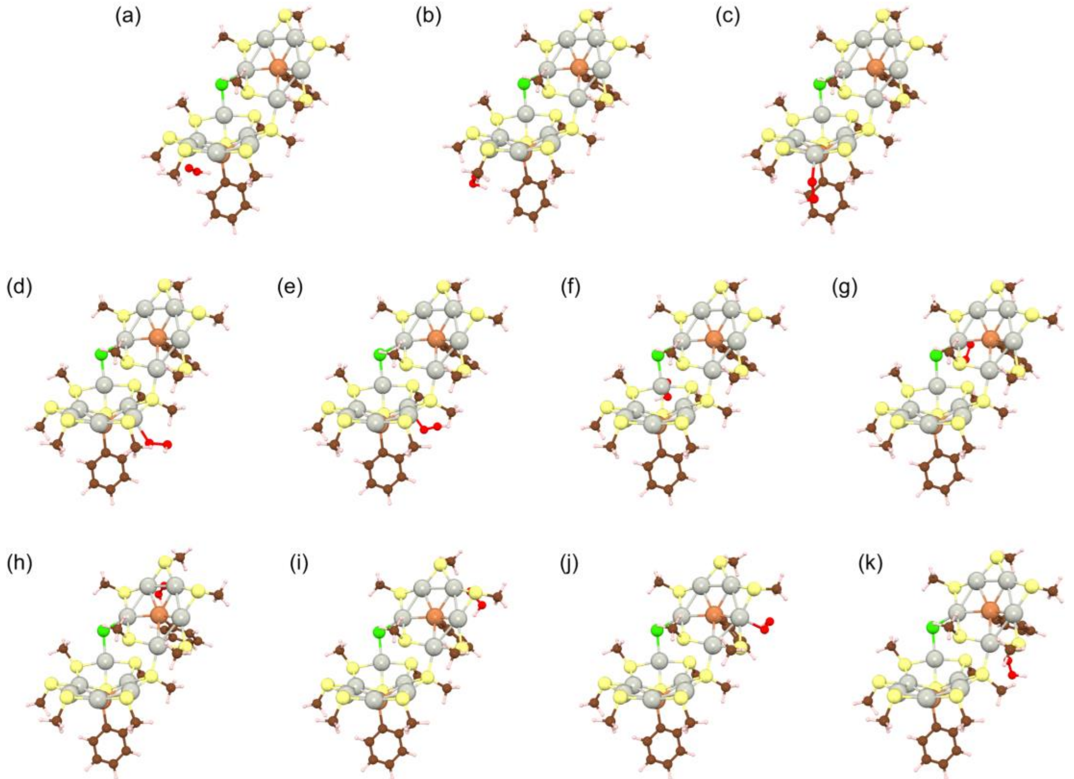


**Fig. S37. Rate-determining steps on possible structural sites of $Pd_{11}$-O clusters.** Pd1 site (a), Pd2 site (b), Pd3 site (c), Pd4 site (d), Pd5 site (e), Pd6 site (f), Pd7 site (g), Pd8 site (h), Pd9 site (i), Pd10 site (j), and Pd11 site (k). Color labels: gray, Pd; yellow, S; orange, Sb; red, O; green, Cl; brown, C; pink, H.

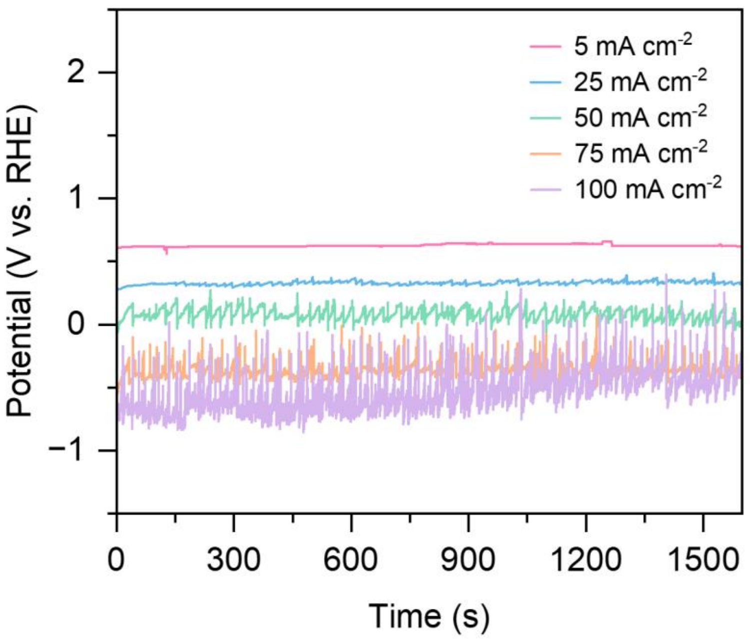


**Fig. S38. Galvanostatic profiles of $Pd_{11}$-C clusters at different current densities.**

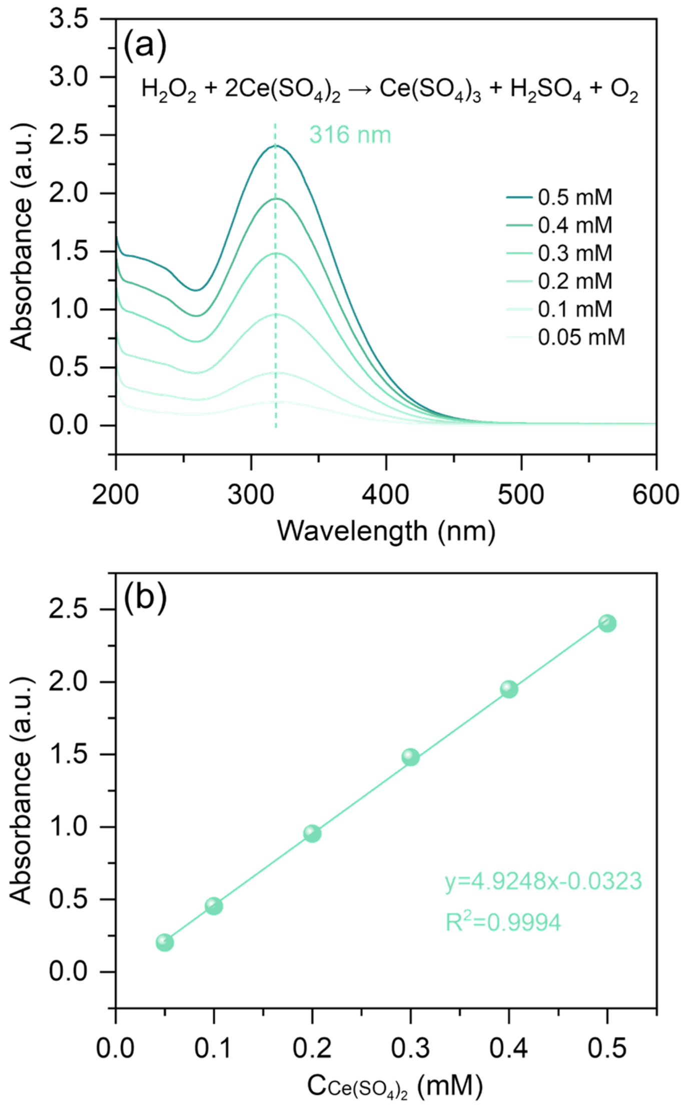


**Fig. S39. Dependence of the absorbance on the $Ce^{4+}$ concentration.** (a) UV–Vis absorption spectra of $Ce(SO_4)_2$ solutions with different concentrations; (b) Linear calibration curve based on absorbance at 316 nm.

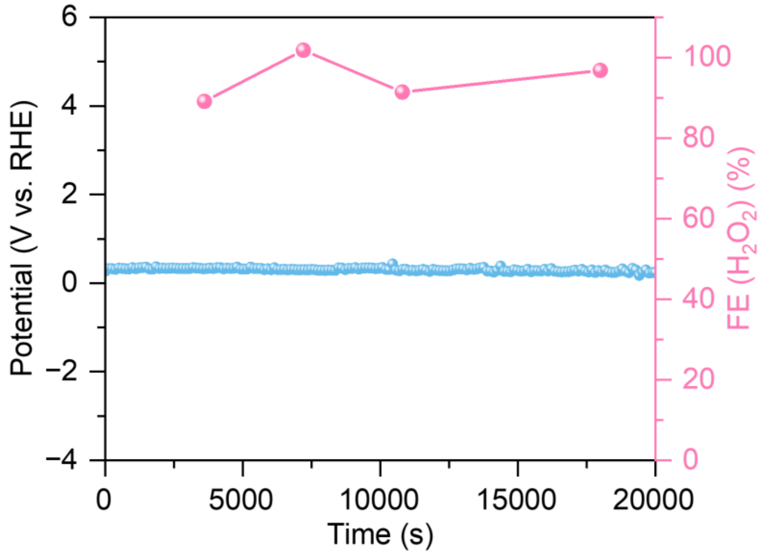


**Fig. S40. Durability test and corresponding $H_2O_2$ FE of $Pd_{11}$-C clusters at 25 mA cm$^{-2}$ in 0.1 M KOH for 20000 s.**

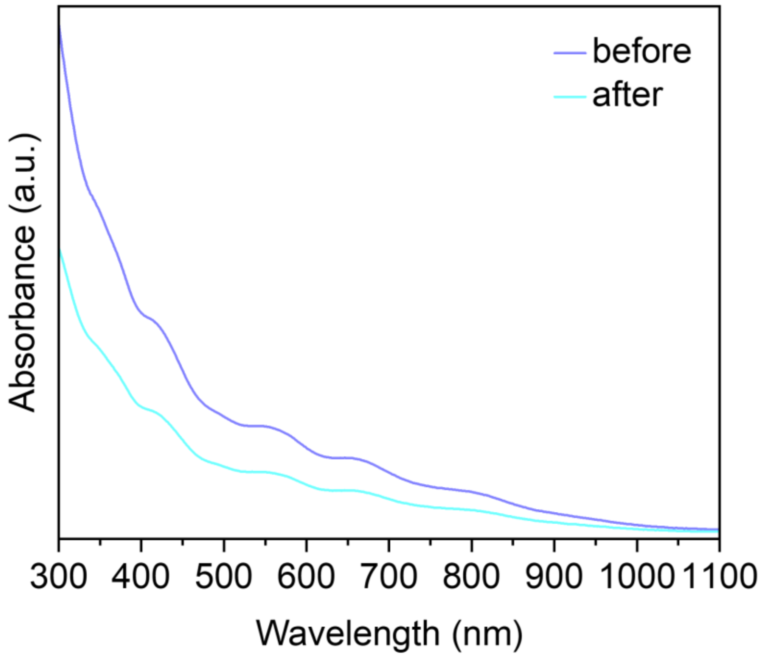


**Fig. S41. UV–Vis–NIR absorption spectra of $Pd_{11}$-C clusters before and after stability test.**

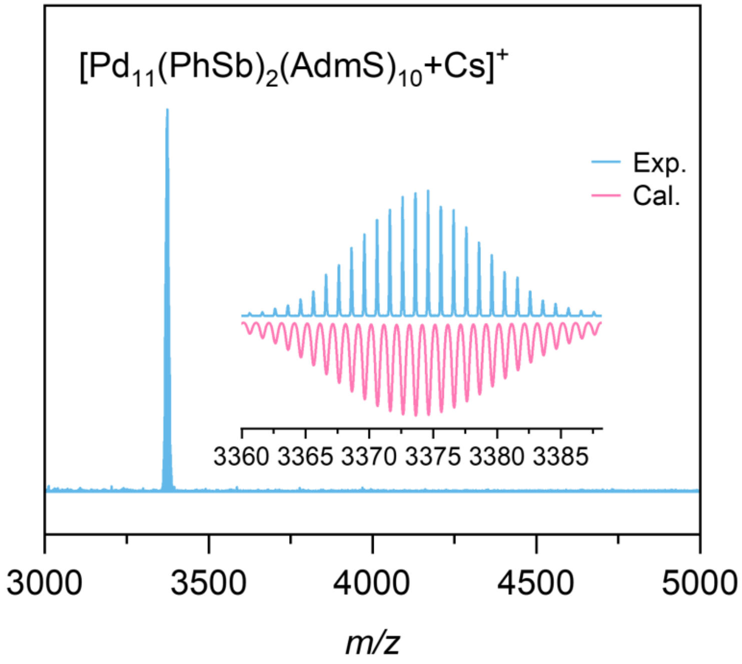


**Fig. S42. ESI-MS spectrum of $Pd_{11}$-C clusters after stability test.**

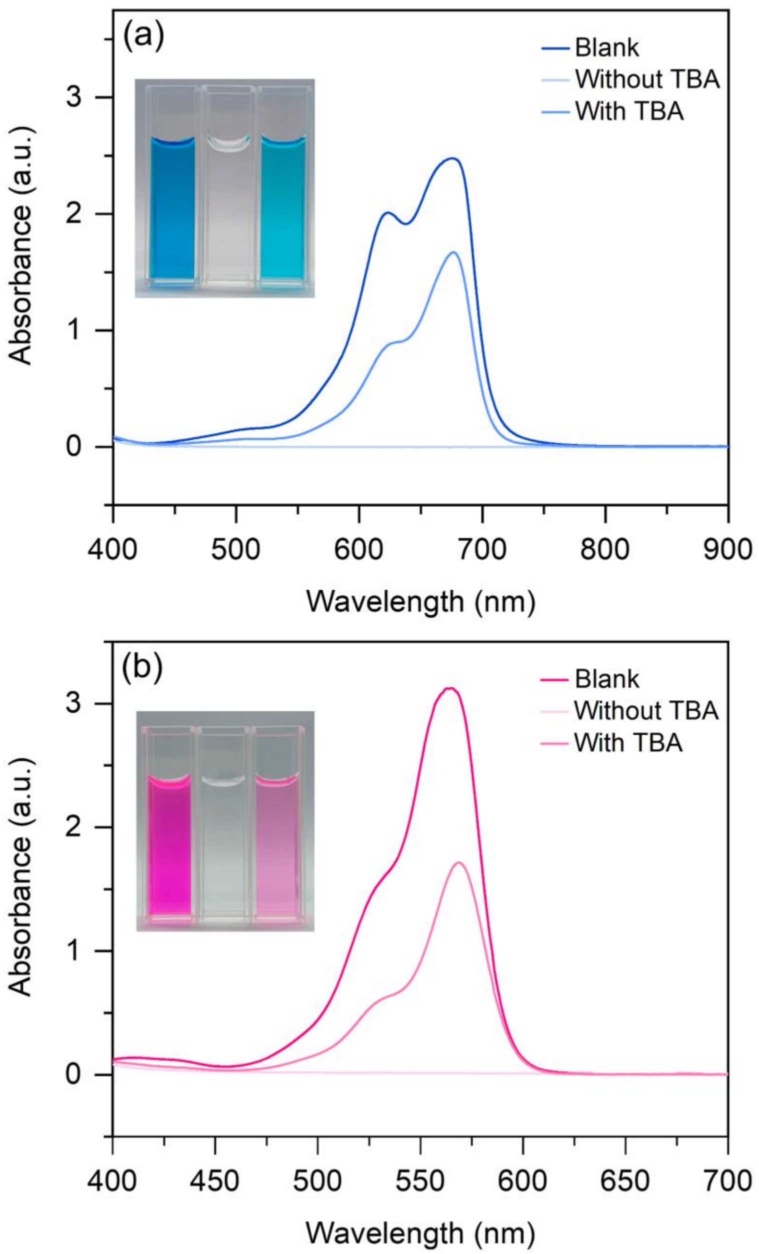


**Fig. S43. Digital images and corresponding UV–Vis absorption spectra of MB (a) and RhB (b) solutions after addition of Fenton's reagent and catholyte, with or without TBA.**

**Table S1. Crystal data and structure refinement for $Pd_{11}$-C.**

| | **$Pd_{11}$-C** |
|---|---|
| Empirical formula | $C_{112}H_{160}Pd_{11}S_{10}Sb_2$ |
| Formula weight | 3240.89 |
| Temperature/K | 193.00 |
| Crystal system | triclinic |
| Space group | *P*-1 |
| a/Å | 16.070(4) |
| b/Å | 16.757(4) |
| c/Å | 26.785(7) |
| α/° | 81.474(15) |
| β/° | 86.876(16) |
| γ/° | 71.347(12) |
| Volume/Å$^3$ | 6758(3) |
| Z | 2 |
| $\rho_{calc}$g/cm$^3$ | 1.593 |
| μ/mm$^{-1}$ | 16.337 |
| F(000) | 3200.0 |
| Radiation | CuKα (λ = 1.54178 Å) |
| 2Θ range for data collection/° | 3.336 to 137.376 |
| Index ranges | $-19 \leq h \leq 15$, $-20 \leq k \leq 20$, $-31 \leq l \leq 32$ |
| Reflections collected | 71476 |
| Independent reflections | 24459 [$R_{int}$ = 0.0875, $R_{sigma}$ = 0.0685] |
| Data/restraints/parameters | 24459/1102/1307 |
| Goodness-of-fit on $F^2$ | 1.024 |
| Final R indexes [I>=2σ (I)] | $R_1$ = 0.0895, $wR_2$ = 0.2376 |
| Final R indexes [all data] | $R_1$ = 0.1100, $wR_2$ = 0.2592 |
| Largest diff. peak/hole / e Å$^{-3}$ | 1.84/-3.09 |

**Table S2. Crystal data and structure refinement for $Pd_{11}$-O.**

| | **$Pd_{11}$-O** |
|---|---|
| Empirical formula | $C_{122}H_{175}ClPd_{11}S_{12}Sb_2{\cdot}(CH_2Cl_2)_4$ |
| Formula weight | 3815.38 |
| Temperature/K | 193.00 |
| Crystal system | triclinic |
| Space group | *P*-1 |
| a/Å | 16.9682(7) |
| b/Å | 19.1269(8) |
| c/Å | 24.8100(10) |
| α/° | 106.467(3) |
| β/° | 92.873(3) |
| γ/° | 97.141(2) |
| Volume/Å$^3$ | 7631.0(6) |
| Z | 2 |
| $\rho_{calc}$g/cm$^3$ | 1.660 |
| μ/mm$^{-1}$ | 16.235 |
| F(000) | 3784.0 |
| Radiation | CuKα (λ = 1.54184 Å) |
| 2Θ range for data collection/° | 4.868 to 136.77 |
| Index ranges | $-20 \le h \le 20$, $-23 \le k \le 23$, $-25 \le l \le 29$ |
| Reflections collected | 80743 |
| Independent reflections | 27700 [$R_{int}$ = 0.0746, $R_{sigma}$ = 0.0796] |
| Data/restraints/parameters | 27700/556/1441 |
| Goodness-of-fit on $F^2$ | 1.060 |
| Final R indexes [I>=2σ (I)] | $R_1$ = 0.1105, $wR_2$ = 0.2840 |
| Final R indexes [all data] | $R_1$ = 0.1173, $wR_2$ = 0.2884 |
| Largest diff. peak/hole / e Å$^{-3}$ | 4.43/-2.42 |

## REFERENCES


1. W.-W. Yang, J.-J. Shen, Multiple heterohelicenes: Synthesis, properties and applications. *Chem. A Eur. J.* **28**, e202202069 (2022).

2. P. Ravat, Carbo[*n*]helicenes restricted to enantiomerize: An insight into the design process of configurationally stable functional chiral PAHs. *Chem. A Eur. J.* **27**, 3957–3967 (2021).

3. M. Gingras, One hundred years of helicene chemistry. Part 1: Non-stereoselective syntheses of carbohelicenes. *Chem. Soc. Rev.* **42**, 968–1006 (2013).

4. M. Gingras, G. Félix, R. Peresutti, One hundred years of helicene chemistry. Part 2: Stereoselective syntheses and chiral separations of carbohelicenes. *Chem. Soc. Rev.* **42**, 1007–1050 (2013).

5. M. Gingras, One hundred years of helicene chemistry. Part 3: Applications and properties of carbohelicenes. *Chem. Soc. Rev.* **42**, 1051–1095 (2013).

6. Y. He, H. Yang, Y. Yue, X. Gan, S. Xiao, X. Chen, S. Zhu, D. Wan, R. He, H. Si, G. Meng, P. Chen, J. Ding, Benzo-extended N^N^N-chelated tetracoordinate boron hetero[8]helicene featuring an inner N–B–N helical rim for circularly polarized TADF. *J. Mater. Chem. C* **13**, 18092–18100 (2025).

7. Y. Shen, C.-F. Chen, Helicenes: Synthesis and applications. *Chem. Rev.* **112**, 1463–1535 (2012).

8. T. Mori, Chiroptical properties of symmetric double, triple, and multiple helicenes. *Chem. Rev.* **121**, 2373–2412 (2021).

9. S.-M. Guo, S. Huh, M. Coehlo, L. Shen, G. Pieters, O. Baudoin, A C–H activation-based enantioselective synthesis of lower carbo[*n*]helicenes. *Nat. Chem.* **15**, 872–880 (2023).

10. Q. Huang, Y.-P. Tang, C.-G. Zhang, Z. Wang, L. Dai, Enantioselective synthesis of helically chiral molecules enabled by asymmetric organocatalysis. *ACS Catal.* **14**, 16256–16265 (2024).

11. D. Sakamoto, I. Gay Sánchez, J. Rybáček, J. Vacek, L. Bednárová, M. Pazderková, R. Pohl, I. Císařová, I. G. Stará, I. Starý, Cycloiridated helicenes as chiral catalysts in the asymmetric transfer hydrogenation of imines. *ACS Catal.* **12**, 10793–10800 (2022).

12. P. Fan, L. Li, D. Qian, Catalytic asymmetric construction of helicenes *via* transformation of biaryls. *Org. Biomol. Chem.* **22**, 3186–3197 (2024).

13. J. Jongkhumkrong, I. Thaveesangsakulthai, W. Sukbangnop, C. Kulsing, T. Sooksimuang, C. Aonbangkhen, S. Sahasithiwat, T. Sriprasart, T. Palaga, N. Chantaravisoot, B. Tomapatanaget, Helicene-hydrazide encapsulated ethyl cellulose as a potential fluorescence sensor for highly specific detection of nonanal in aqueous solutions and a proof-of-concept clinical study in lung fluid. *ACS Appl. Mater. Interfaces* **14**, 49495–49507 (2022).

14. A. Petdum, N. Faichu, J. Sirirak, P. Khammultri, V. Promarak, W. Panchan, T. Sooksimuang, A. Charoenpanich, N. Wanichacheva, [5]Helicene-rhodamine 6 G hybrid-based sensor for ultrasensitive $Hg^{2+}$ detection and its biological applications. *J. Photochem. Photobiol. A Chem.* **394**, 112473 (2020).

15. P. Patawanich, A. Petdum, J. Sirirak, K. Chatree, A. Charoenpanich, W. Panchan, K. Setthakarn, A. Kamkaew, T. Sooksimuang, P. Maitarad, N. Wanichacheva, Highly selective zinc(II) triggered "Turn-ON" [5]helicene-based fluorescence sensor: Its application in liver and brain cells imaging. *J. Mol. Liq.* **362**, 119710 (2022).

16. J. Feng, L. Wang, X. Xue, Z. Chao, B. Hong, Z. Gu, Ring-expansion strategy for α-aryl azahelicene construction: Building blocks for optoelectronic materials. *Org. Lett.* **23**, 8056–8061 (2021).

17. S. D. Dongre, G. Venugopal, V. Kumar, A. B. Jadhav, J. Kumar, S. S. Babu, Chiroptical amplification of [7]-helicene nanographene by additional helical chirality. *Angew. Chem. Int. Ed. Engl.* **64**, e202420767 (2025).

18. K. Kovida, J. Malinčík, T. Šolomek, Effect of π-electron conjugation on the chiroptical properties of helicene carbon nanohoops. *Helv. Chim. Acta* **108**, e202400166 (2025).

19. C. Maeda, Y. Daigen, S. Michishita, T. Ema, Pyrene-fused azahelicene showing circularly polarized luminescence. *Org. Lett.* **27**, 6648–6653 (2025).

20. D. Sakamaki, S. Tanaka, K. Tanaka, M. Takino, M. Gon, K. Tanaka, T. Hirose, D. Hirobe, H. M. Yamamoto, H. Fujiwara, Double heterohelicenes composed of benzo[*b*]- and dibenzo[*b*,*i*]phenoxazine: A comprehensive comparison of their electronic and chiroptical properties. *J. Phys. Chem. Lett.* **12**, 9283–9292 (2021).

21. A. Mondal, R. Toyoda, R. Costil, B. L. Feringa, Chemically driven rotatory molecular machines. *Angew. Chem. Int. Ed. Engl.* **61**, e202206631 (2022).

22. Y. Gisbert, M. Ovalle, C. N. Stindt, R. Costil, B. L. Feringa, Coupling rotary motion to helicene inversion within a molecular motor. *Angew. Chem. Int. Ed. Engl.* **64**, e202416097 (2025).

23. J. Meisenheimer, K. Witte, Reduction von 2-Nitronaphtalin. *Chem. Ber.* **36**, 4153–4164 (1903).

24. M. Toya, T. Omine, F. Ishiwari, A. Saeki, H. Ito, K. Itami, Expanded [2,1][*n*]carbohelicenes with 15- and 17-benzene rings. *J. Am. Chem. Soc.* **145**, 11553–11565 (2023).

25. E. González-Fernández, L. D. M. Nicholls, L. D. Schaaf, C. Farès, C. W. Lehmann, M. Alcarazo, Enantioselective synthesis of [6]carbohelicenes. *J. Am. Chem. Soc.* **139**, 1428–1431 (2017).

26. L. Zhang, S. Chen, J. Jiang, X. Dong, Y. Cai, H.-J. Zhang, J. Lin, Y.-B. Jiang, *C*- and *S*-shaped perylene diimide heterohelicenes: Modular synthesis and spiral-stair-like π-stacking. *Org. Lett.* **24**, 3179–3183 (2022).

27. Z. Sun, C. Yu, N. Zhang, L. Li, Y. Jiao, P. Thiruvengadam, D. Wu, F. Zhang, Divergent synthesis of double heterohelicenes as strong chiral double hydrogen-bonding donors. *Org. Lett.* **24**, 6670–6675 (2022).

28. W. Zhuang, Y. Liu, Z. Deng, Y. Guo, P. C. Y. Chow, D. Lee Phillips, W. Jiang, Z. Wang, J. Liu, Synthesis, structures, and chiroptical properties of NBN-doped helicenes with boron atoms in the inner rims. *Precis. Chem.* **2**, 28–39 (2024).

29. Z. Zhou, Y. Zheng, M. Wang, Q. Li, H. Guo, G. Zhou, Controlling of enantiomeric and phosphorescent behaviors in symmetric and unsymmetric S-shaped double helicenes containing thiophene and/or thiopyran rings. *Org. Lett.* **27**, 8112–8117 (2025).

30. H.-C. Huang, Y.-C. Hsieh, P.-L. Lee, C.-C. Lin, Y.-S. Ho, W.-K. Shao, C.-T. Hsieh, M.-J. Cheng, Y.-T. Wu, Highly distorted multiple helicenes: Syntheses, structural analyses, and properties. *J. Am. Chem. Soc.* **145**, 10304–10313 (2023).

31. L. Dang, W. Xu, S. Qiu, Y. Yu, Z. Ma, L. Yue, H. Su, C. Li, H. Wang, Construction and circularly polarized luminescence of thiophene-based multiple helicenes. *Org. Lett.* **26**, 10141–10145 (2024).

32. C.-C. Lin, M.-L. Pan, P.-L. Li, W.-T. Ou, M.-J. Cheng, Y.-T. Wu, Syntheses, structural analyses, and properties of condensed arenes with multihelicity. *Org. Lett.* **26**, 7847–7852 (2024).

33. E. Anger, M. Rudolph, C. Shen, N. Vanthuyne, L. Toupet, C. Roussel, J. Autschbach, J. Crassous, R. Réau, From hetero- to homochiral bis(metallahelicene)s based on a $Pt^{III}$–$Pt^{III}$ bonded scaffold: Isomerization, structure, and chiroptical properties. *J. Am. Chem. Soc.* **133**, 3800–3803 (2011).

34. E. Anger, M. Rudolph, L. Norel, S. Zrig, C. Shen, N. Vanthuyne, L. Toupet, J. A. G. Williams, C. Roussel, J. Autschbach, J. Crassous, R. Réau, Multifunctional and reactive enantiopure organometallic helicenes: Tuning chiroptical properties by structural variations of mono- and bis(platinahelicene)s. *Chem. A Eur. J.* **17**, 14178–14198 (2011).

35. L. Chi, Y. Liu, M. Wang, Q. Xu, Vibronic coupling and multiple electronic states effect in ABS and ECD spectra: Three [7]helicene derivatives. *J. Phys. Chem. A* **129**, 1051–1059 (2025).

36. M. Vanzan, S. Bertuletti, G. Becatti, B. Bazan, M. T. Rispens, S. I. C. Wan, M. Leeman, W. L. Noorduin, F. Baletto, A rational framework to estimate the chiroptical activity of [6]helicene derivatives. *J. Phys. Chem. A* **129**, 9537–9547 (2025).

37. S. C. Sevov, J. D. Corbett, Carbon-free fullerenes: Condensed and stuffed anionic examples in indium systems. *Science* **262**, 880–883 (1993).

38. M. J. Moses, J. C. Fettinger, B. W. Eichhorn, Interpenetrating $As_{20}$ fullerene and $Ni_{12}$ icosahedra in the onion-skin $[As@Ni_{12}@As_{20}]^{3-}$ ion. *Science* **300**, 778–780 (2003).

39. S. Stegmaier, T. F. Fässler, A bronze matryoshka: The discrete intermetalloid cluster $[Sn@Cu_{12}@Sn_{20}]^{12-}$ in the ternary phases $A_{12}Cu_{12}Sn_{21}$ (A = Na, K). *J. Am. Chem. Soc.* **133**, 19758–19768 (2011).

40. Z. Li, H. Ruan, L. Wang, C. Liu, L. Xu, Counterion-induced crystallization of intermetalloid *Matryoshka* clusters $[Sb@Pd_{12}@Sb_{20}]^{3-,4-}$. *Dalton Trans.* **46**, 3453–3456 (2017).

41. Y.-H. Xu, W.-J. Tian, A. Muñoz-Castro, G. Frenking, Z.-M. Sun, An all-metal fullerene: $[K@Au_{12}Sb_{20}]^{5-}$. *Science* **382**, 840–843 (2023).

42. C.-C. Shu, D. W. Szczepanik, A. Muñoz-Castro, M. Solà, Z.-M. Sun, $[K_2(Bi@Pd_{12}@Bi_{20})]^{4-}$: An endohedral inorganic fullerene with spherical aromaticity. *J. Am. Chem. Soc.* **146**, 14166–14173 (2024).

43. A. Müller, E. Krickemeyer, H. Bögge, M. Schmidtmann, F. Peters, Organizational forms of matter: An inorganic super fullerene and keplerate based on molybdenum oxide. *Angew. Chem. Int. Ed. Engl.* **37**, 3359–3363 (1998).

44. J. Bai, A. V. Virovets, M. Scheer, Synthesis of inorganic fullerene-like molecules. *Science* **300**, 781–783 (2003).

45. S.-F. Yuan, C.-Q. Xu, J. Li, Q.-M. Wang, A ligand-protected golden fullerene: The dipyridylamido $Au_{32}{}^{8+}$ nanocluster. *Angew. Chem. Int. Ed. Engl.* **58**, 5906–5909 (2019).

46. L. Tang, W. Dong, Q. Han, B. Wang, Z. Wu, S. Wang, Structure and optical properties of an $Ag_{135}Cu_{60}$ nanocluster incorporating an $Ag_{135}$ buckminsterfullerene-like topology. *Nat. Synth.* **4**, 506–513 (2025).

47. Q. You, X.-L. Jiang, W. Fan, Y.-S. Cui, Y. Zhao, S. Zhuang, W. Gu, L. Liao, C.-Q. Xu, J. Li, Z. Wu, $Pd_8$ nanocluster with nonmetal-to-metal- ring coordination and promising photothermal conversion efficiency. *Angew. Chem. Int. Ed. Engl.* **63**, e202313491 (2024).

48. B. Mondal, P. S. Mukherjee, Cage encapsulated gold nanoparticles as heterogeneous photocatalyst for facile and selective reduction of nitroarenes to azo compounds. *J. Am. Chem. Soc.* **140**, 12592–12601 (2018).

49. R. McCaffrey, H. Long, Y. Jin, A. Sanders, W. Park, W. Zhang, Template synthesis of gold nanoparticles with an organic molecular cage. *J. Am. Chem. Soc.* **136**, 1782–1785 (2014).

50. R. Tao, K. Kang, X. Li, R. Li, R. Huang, Y. Jin, L. Qiu, W. Zhang, Controlled synthesis of palladium nanoparticles with size-dependent catalytic activities enabled by organic molecular cages. *Inorg. Chem.* **60**, 12517–12525 (2021).

51. J. Tang, K. Jia, R. Zhang, C. Liu, X. Lin, T. Ge, X. Liu, Q. Zhao, W. Liu, D. Ma, H. Fan, J. Huang, Selective hydrogenation of alkyne by atomically precise $Pd_6$ nanocluster catalysts: Accurate construction of the coplanar and specific active sites. *ACS Catal.* **14**, 2463–2472 (2024).

52. S. K. Eswaramoorthy, A. Dass, Atomically precise palladium nanoclusters with 21 and 38 Pd atoms protected by phenylethanethiol. *J. Phys. Chem. C* **126**, 444–450 (2022).

53. L. Luo, Z. Liu, X. Du, R. Jin, Near-infrared dual emission from the $Au_{42}(SR)_{32}$ nanocluster and tailoring of intersystem crossing. *J. Am. Chem. Soc.* **144**, 19243–19247 (2022).

54. W. Wei, Y. Lu, W. Chen, S. Chen, One-pot synthesis, photoluminescence, and electrocatalytic properties of subnanometer-sized copper clusters. *J. Am. Chem. Soc.* **133**, 2060–2063 (2011).

55. Q. You, X.-L. Jiang, Y. Zhao, W. Gu, J. Li, Z. Wu, Inverse palladocenes. *Nat. Commun.* **17**, 2171 (2026).

56. J. R. Shallenberger, Determination of chemistry and microstructure in $SiO_x$ ($0.1<x<0.8$) films by x-ray photoelectron spectroscopy. *J. Vac. Sci. Technol. A* **14**, 693–698 (1996).

57. D. S. Jensen, S. S. Kanyal, N. Madaan, M. A. Vail, A. E. Dadson, M. H. Engelhard, M. R. Linford, Silicon (100)/$SiO_2$ by XPS. *Surf. Sci. Spectra* **20**, 36–42 (2013).

58. S. Zhuang, D. Chen, Q. You, W. Fan, J. Yang, Z. Wu, Thiolated, reduced palladium nanoclusters with resolved structures for the electrocatalytic reduction of oxygen. *Angew. Chem. Int. Ed. Engl.* **61**, e202208751 (2022).

59. X. Tang, H. Shen, H. Huang, L. Li, F. Luo, G. Tian, H. Deng, B. K. Teo, N. Zheng, A versatile strategy for the controlled synthesis of atomically precise palladium nanoclusters. *Small Methods* **9**, e2400040 (2025).

60. S. L. Benjamin, T. Krämer, W. Levason, M. E. Light, S. A. Macgregor, G. Reid, $[Pd_4(\mu_3\text{-}SbMe_3)_4(SbMe_3)_4]$: A Pd(0) tetrahedron with $\mu_3$-bridging trimethylantimony ligands. *J. Am. Chem. Soc.* **138**, 6964–6967 (2016).

61. M. S. Newman, D. Lednicer, The synthesis and resolution of hexahelicene. *J. Am. Chem. Soc.* **78**, 4765–4770 (1956).

62. K. Dhbaibi, L. Favereau, J. Crassous, Enantioenriched helicenes and helicenoids containing main-group elements (B, Si, N, P). *Chem. Rev.* **119**, 8846–8953 (2019).

63. T.-S. Zhang, W. Fei, N. Li, Y. Zhang, C. Xu, Q. Luo, M.-B. Li, Open nitrogen site-induced kinetic resolution and catalysis of a gold nanocluster. *Nano Lett.* **23**, 235–242 (2023).

64. J. Chai, S. Yang, T. Chen, Q. Li, S. Wang, M. Zhu, Chiral inversion and conservation of clusters: a case study of racemic $Ag_{32}Cu_{12}$ nanocluster. *Inorg. Chem.* **60**, 9050–9056 (2021).

65. J. B. Patty, S. Havenridge, D. Tietje-Mckinney, M. A. Siegler, K. K. Singh, R. Hajy Hosseini, M. Ghabin, C. M. Aikens, A. Das, Crystal structure and optical properties of a chiral mixed thiolate/stibine-protected $Au_{18}$ cluster. *J. Am. Chem. Soc.* **144**, 478–484 (2022).

66. Z. Fallah, S. Malola, M. F. Matus, H. Häkkinen, Chiral ligand-protected gold nanoclusters as biosensors for small chiral biomolecules: A computational study. *ACS Nano* **20**, 7743 – 7751 (2026).

67. Y.-Q. Ding, Z.-Y. Chen, Z.-Y. Li, X. Cheng, M. Wang, J.-B. Ma, Lithium-assisted dinitrogen reduction mediated by $Nb_2LiNO_{1–4}^-$ cluster anions: Electron donors or structural units. *J. Phys. Chem. A* **126**, 1511–1517 (2022).

68. Z. Liang, H. Guo, G. Zhou, K. Guo, B. Wang, H. Lei, W. Zhang, H. Zheng, U.-P. Apfel, R. Cao, Metal–organic-framework-supported molecular electrocatalysis for the oxygen reduction reaction. *Angew. Chem. Int. Ed. Engl.* **60**, 8472–8476 (2021).

69. P. Xia, T. He, Y. Sun, X. Duan, X. Chen, Z.-S. Zhu, C. Wang, Y. Liu, Q. He, Z. Ye, Defective-engineered ZnO encapsulated in N-doped carbon for sustainable $2e^-$ ORR: Interfacial Zn–N bond regulated oxygen reduction pathways. *ACS Catal.* **14**, 12917–12927 (2024).

70. C. Mu, B. Wang, Q. Yao, Q. He, J. Xie, Composition-dependent catalytic performance of $Au_xAg_{25-x}$ alloy nanoclusters for oxygen reduction reaction. *Nano Res.* **17**, 9490–9497 (2024).

71. X. Hu, Y. Sun, Y. Chen, F. Liu, X. Lei, S. Wang, Q. Hu, Z. Wang, Z. Gan, X. Zheng, D. Sun, Customizing ferrocene units into atomically precise $Cu_{11}$ clusters for boosting oxygen reduction to $H_2O_2$. *J. Am. Chem. Soc.* **148**, 15426–15436 (2026).

72. S. Siahrostami, A. Verdaguer-Casadevall, M. Karamad, D. Deiana, P. Malacrida, B. Wickman, M. Escudero-Escribano, E. A. Paoli, R. Frydendal, T. W. Hansen, I. Chorkendorff, I. E. L. Stephens, J. Rossmeisl, Enabling direct $H_2O_2$ production through rational electrocatalyst design. *Nat. Mater.* **12**, 1137–1143 (2013).

73. G. Zhu, S. Zhao, Y. Yu, X. Fan, K. Liu, X. Quan, Y. Liu, Tuning local proton concentration and *OOH intermediate generation for efficient acidic $H_2O_2$ electrosynthesis at ampere-level current density. *Angew. Chem. Int. Ed. Engl.* **64**, e202503626 (2025).

74. B. Hammer, J. K. Nørskov, Theoretical surface science and catalysis—Calculations and concepts. *Adv. Catal.* **45**, 71–129 (2000).

75. C. Pan, Y. Jiao, A. B. Kersting, M. Zavarin, Plutonium redox transformation in the presence of iron, organic matter, and hydroxyl radicals: Kinetics and mechanistic insights. *Environ. Sci. Technol.* **55**, 1800–1810 (2021).

76. K. Yang, B. Jia, J. Liu, K. Zhu, J. Qin, H. Jia, A novel perspective on the role of hydroxyl radicals in soil organic carbon mineralization within the detritusphere: Stimulating C-degrading enzyme activities. *Environ. Sci. Technol.* **59**, 5045–5055 (2025).

77. M. J. Frisch, G. W. Trucks, H. B. Schlegel, G. E. Scuseria, M. A. Robb, J. R. Cheeseman, G. Scalmani, V. Barone, G. A. Petersson, H. Nakatsuji, X. Li, M. Caricato, A. V. Marenich, J. Bloino, B. G. Janesko, R. Gomperts, B. Mennucci, H. P. Hratchian, J. V. Ortiz, A. F. Izmaylov, J. L. Sonnenberg, D. Williams-Young, F. Ding, F. Lipparini, F. Egidi, J. Goings, B. Peng, A. Petrone, T. Henderson, D. Ranasinghe, V. G. Zakrzewski, J. Gao, N. Rega, G. Zheng, W. Liang, M. Hada, M. Ehara, K. Toyota, R. Fukuda, J. Hasegawa, M. Ishida, T. Nakajima, Y. Honda, O. Kitao, H. Nakai, T. Vreven, K. Throssell, J. A. Montgomery, Jr., J. E. Peralta, F. Ogliaro, M. J. Bearpark, J. J. Heyd, E. N. Brothers, K. N. Kudin, V. N. Staroverov, T. A. Keith, R. Kobayashi, J. Normand, K. Raghavachari, A. P. Rendell, J. C. Burant, S. S. Iyengar, J. Tomasi, M. Cossi, J. M. Millam, M. Klene, C. Adamo, R. Cammi, J. W. Ochterski, R. L. Martin, K. Morokuma, O. Farkas, J. B. Foresman, D. J. Fox, Gaussian 16, revision B.01 (Gaussian Inc., Wallingford, CT, 2016).

78. J. P. Perdew, K. Burke, M. Ernzerhof, Generalized gradient approximation made simple. *Phys. Rev. Lett.* **77**, 3865–3868 (1996).

79. J. P. Perdew, K. Burke, M. Ernzerhof, Errata: Generalized gradient approximation made simple. *Phys. Rev. Lett.*, **78**, 1396 (1997).

80. C. Adamo, V. Barone, Toward reliable density functional methods without adjustable parameters: The PBE0 model. *J. Chem. Phys.* **110**, 6158–6170 (1999).

81. S. Grimme, S. Ehrlich, L. Goerigk, Effect of the damping function in dispersion corrected density functional theory. *J. Comput. Chem.* **32**, 1456–1465 (2011).

82. F. Weigend, R. Ahlrichs, Balanced basis sets of split valence, triple zeta valence and quadruple zeta valence quality for H to Rn: Design and assessment of accuracy. *Phys. Chem. Chem. Phys.* **7**, 3297–3305 (2005).

83. T. Lu, F. Chen, Multiwfn: A multifunctional wavefunction analyzer. *J. Comput. Chem.* **33**, 580–592 (2012).

84. A. E. Reed, F. Weinhold, Natural bond orbital analysis of near-Hartree–Fock water dimer. *J. Chem. Phys.* **78**, 4066–4073 (1983).

85. A. E. Reed, R. B. Weinstock, F. Weinhold, Natural population analysis. *J. Chem. Phys.* **83**, 735–746 (1985).

86. D. Y. Zubarev, A. I. Boldyrev, Developing paradigms of chemical bonding: Adaptive natural density partitioning. *Phys. Chem. Chem. Phys.* **10**, 5207–5217 (2008).

87. T. Lu, A comprehensive electron wavefunction analysis toolbox for chemists, Multiwfn. *J. Chem. Phys.* **161**, 082503(2024).

88. G. Kresse, J. Furthmüller, Efficiency of ab-initio total energy calculations for metals and semiconductors using a plane-wave basis set. *Comput. Mater. Sci.* **6**, 15–50 (1996).

89. G. Kresse, J. Furthmüller, Efficient iterative schemes for *ab initio* total-energy calculations using a plane-wave basis set. *Phys. Rev. B* **54**, 11169–11186 (1996).

90. G. Kresse, D. Joubert, From ultrasoft pseudopotentials to the projector augmented-wave method. *Phys. Rev. B* **59**, 1758–1775 (1999).

91. H. J. Monkhorst, J. D. Pack, Special points for Brillouin-zone integrations. *Phys. Rev. B* **13**, 5188–5192 (1976).
